# Blood lipids and the risk of aortic aneurysm: results from the UK Biobank study and a systematic review and meta-analysis of cohort studies

**DOI:** 10.1007/s10654-025-01344-4

**Published:** 2026-01-24

**Authors:** Estelle Ioannidou, Dagfinn Aune, Ioannis Theodosopoulos, Alicia K. Heath

**Affiliations:** 1 Department of Epidemiology and Biostatistics, School of Public Health, Imperial College London, White City Campus, 90 Wood Lane, London, W12 0BZ UK; 2 Moorfields Eye Hospital, London, UK; 3 Department of Nutrition, Oslo New University College, Oslo, Norway; 4 Department of Research, Cancer Registry of Norway, Norwegian Institute of Public Health, Oslo, Norway; 5 Department of Vascular Surgery, Attikon, University Hospital of Athens, Athens, Greece

**Keywords:** Aortic aneurysm, Systematic review, Meta-analysis, Lipids, Cholesterol

## Abstract

Circulating lipids are potentially modifiable risk factors for aortic aneurysm, however, associations between levels of specific lipids and aortic aneurysm are unclear. We analysed associations between blood lipids and aortic aneurysm in the UK Biobank and summarized the available data in a systematic review and meta-analysis of published cohort studies. Up to 429,610 participants aged 38–73 years at baseline in 2006–2010 in UK Biobank were included. Multivariable Cox regression was used to estimate hazard ratios (HRs) and 95% confidence intervals (CIs) for the associations between serum/plasma lipids (total cholesterol, low-density lipoprotein [LDL] cholesterol, high-density lipoprotein [HDL] cholesterol, triglycerides, apolipoprotein A1, apolipoprotein B, and lipoprotein(a)) and risk of aortic aneurysm. PubMed and Embase databases were searched up to November 11th, 2025, to identify relevant cohort studies. Random-effects models were used to estimate summary relative risks (RRs) and 95% CIs for the associations between blood lipid levels and aortic aneurysm risk. In UK Biobank, 2,434 aortic aneurysm cases occurred during 12.3 years of follow-up. Positive associations were observed between the highest vs. lowest quintile of total cholesterol (HR 1.22, 95% CI 1.07–1.41), LDL cholesterol (1.39, 1.21–1.60), apolipoprotein B (1.52, 1.33–1.74), non-HDL cholesterol (1.50, 1.29–1.73), triglycerides (1.23, 1.06–1.42), lipoprotein(a) (1.34, 1.17–1.54) and aortic aneurysm risk, while HDL cholesterol (0.57, 0.48–0.67) and apolipoprotein A1 (0.54, 0.46–0.63) were inversely associated. Eighteen cohort studies were included in the meta-analysis. The summary RR (95% CI) per 1 mmol/L increase was 1.16 (1.10–1.22, I^2^ = 74%, *n* = 15) for total cholesterol, 1.11 (1.04–1.19,I^2^ = 63%, *n* = 4) for LDL cholesterol, 1.28 (0.98–1.67, I^2^ = 89%, *n* = 2) for non-HDL cholesterol, 0.59 (0.50–0.70, I^2^ = 72%, *n* = 5) for HDL cholesterol, 1.02 (0.95–1.10, I^2^ = 55%, *n* = 7) for triglycerides, and 1.34 (1.17–1.55, I^2^ = 57%, *n* = 4) per 50 mg/dl incrememt in lipoprotein(a). Additional positive (apolipoprotein B, non-HDL cholesterol) and inverse (apolipoprotein A1) associations were observed in high vs. low meta-analyses. These findings suggest higher levels of total cholesterol, LDL cholesterol, apolipoprotein B, non-HDL cholesterol, lipoprotein(a), and possibly triglycerides are associated with increased risk of aortic aneurysm, while higher levels of HDL cholesterol and apolipoprotein A1 are associated with lower risk.

**Supplementary Information:**

The online version contains supplementary material available at 10.1007/s10654-025-01344-4.

## Introduction

The prevalence of aortic aneurysm is currently increasing due to population aging worldwide and constitutes a growing threat to public health [1–5]. In 2015, 168,000 deaths and 2.9 million disability adjusted life years globally were attributed to aortic aneurysms [6]. The vast majority (80–90%) of aortic aneurysms are found in the abdominal segment of the aorta, while other types include thoracic and thoracoabdominal aortic aneurysms (2). An abdominal aortic aneurysm is a permanent localised dilation of the aorta, involving all layers of the artery wall, exceeding the normal segment diameter by more than 50% or > 3 cm [7, 8]. Alternative definitions include a maximal aortic diameter of 4 cm or an infrarenal to suprarenal diameter ratio of more than 1.2 [9]. Abdominal aortic aneurysms are usually located in the infrarenal segment of the aorta [1, 9]. Abdominal aortic aneurysms are most common in men aged over 65 years and around 1% of deaths among older men in developed countries are attributed to a ruptured abdominal aortic aneurysm, making it the tenth commonest cause of mortality [10]. The mortality of a ruptured aortic aneurysm can be as high as 60–80% whereas it can be minimised to 3–7% if the aneurysm is identified before it ruptures and is repaired with surgical intervention [11]. Increasing our understanding of the disease’s pathophysiology, as well as the risk factors associated with it could facilitate primary prevention, however, apart from smoking, elevated blood pressure and low physical activity [12–15], few modifiable risk factors for aortic aneurysms have been established. Traumatic, hereditary (Marfan’s and Ehlers-Danlos syndromes) and infectious causes, as well as anomalies of the connective tissue metabolism, have also been suggested as risk factors [1].

Several biological pathways are involved in the development of aortic aneurysms, including oxidative stress, inflammation, apoptosis of smooth muscle cells, degradation of elastin, collagen, and glycosaminoglycans in the aorta by matrix metalloproteinases, and the renin-angiotensin system [16]. There is also evidence that atherosclerosis is involved in aortic aneurysm aetiology [8, 17], which suggests levels of circulating lipids might also play a role, however, the associations are currently unclear. Several cohort studies have suggested higher plasma total cholesterol is associated with increased risk of aortic aneurysm [3, 8, 18–21, 23, 24], however, not all studies were consistent [22, 25]. In addition, some studies have suggested that higher high density lipoprotein (HDL) cholesterol may be associated with lower aortic aneurysm risk, however, the strength of the observed associations differed somewhat between studies [3, 19, 21, 26]. Studies on triglycerides and aortic aneurysm have shown mixed results [3, 8, 18, 20, 26, 27], while studies on low-density lipoprotein (LDL) cholesterol [3, 27], and lipoprotein(a) [5, 28] and aortic aneurysm risk have been more limited, but suggested positive associations. Establishing whether blood lipids are related to aortic aneurysm risk could be important for the prevention of the disease by identifying targets for intervention through modification of diet and lifestyle factors as well as medication use.

Based on this, we analysed the association between blood lipid concentrations and aortic aneurysm risk in the UK Biobank study and conducted a systematic review and meta-analysis to summarise all published evidence on the associations between blood concentrations of lipids (total cholesterol, LDL cholesterol, HDL cholesterol, triglycerides, lipoprotein(a) or apolipoprotein A1, apolipoprotein B) and subsequent risk of aortic aneurysm.

## Methods

### UK Biobank

The UK Biobank study is a cohort of 0.5 million British residents aged 37–73 years at baseline in 2006–2010 [29]. The UK Biobank has ethical approval from the North West Multi-Centre Research Ethics Committee, the National Information Governance Board for Health and Social Care in England and Wales, and the Community Health Index Advisory Group in Scotland. In addition, an independent Ethics and Governance Council was formed in 2004 to oversee UK Biobank’s continuous adherence to the Ethics and Governance framework, which were developed for the study [29]. All participants provided written informed consent [29].

Biological samples (blood, urine, saliva), anthropometric measures, and detailed touchscreen data on diet, physical activity, smoking, alcohol consumption, socio-demographic characteristics, and medical history were collected from the participants. Linkages to National Health Service (NHS) records provided information on aortic aneurysm hospitalisations and deaths. International Classification of Diseases version 10 (ICD-10) was used to identify incident and fatal cases of aortic aneurysm using codes I71.1-I71.9. Aortic aneurysm subtypes were classified as follows: thoracic aortic aneurysm (I71.1-I71.2), abdominal aortic aneurysm (I71.3-I71.4), thoracoabdominal aortic aneurysm (I71.5-I71.6), and unspecified site aortic aneurysm (I71.8-I71.9), ruptured aortic aneurysm (I71.1, I71.3, I71.5, I71.8), and non-ruptured aortic aneurysm (I71.2, I71.4, I71.6, I71.9). For participants who died from aortic aneurysm, but with no previous hospital record of the disease, we used date of death as date of occurrence. Participants were followed from the date of the baseline visit to the date of aortic aneurysm hospitalization or death, death from any other cause, loss to follow-up, or date of censoring (30th September 2021 for England and Wales and 31st October 2021 for Scotland), whichever occurred first. The current dataset included 501,941 participants before exclusions were made for prevalent disease and missing data. For the current analysis, we excluded individuals with prevalent aortic aneurysm at baseline (*n* = 476), different self-reported compared to genetic sex (*n* = 370), missing BMI (*n* = 3,094), missing smoking status (*n* = 2,422), missing measurements of total cholesterol (*n* = 31,386), low-density lipoprotein cholesterol (*n* = 990), triglycerides (*n* = 266), apolipoprotein B (*n* = 2,366), and those with prevalent ischemic heart disease or stroke at baseline (*n* = 30,961), leaving 429,610 participants for the analyses of these blood lipids. Further exclusions were made for missing measurements of HDL cholesterol (*n* = 36,198), apolipoprotein A1 (*n* = 38,517), and lipoprotein(a) (*n* = 84,652) in those specific analyses, leaving 393,412, 391,093, and 344,958 participants for the respective analyses. Non-HDL cholesterol was calculated by subtracting HDL cholesterol from total cholesterol and included the same number of participants as the analysis of HDL cholesterol (*n* = 393,412). Lipoprotein(a) was converted from nmol/L to mg/dl using the formula (Lp(a) [in nmol/L] + 3.83) / 2.18 = Lp(a) [in mg/dl] [30].

### Statistical analysis

Multivariable Cox proportional hazards regression models were used to estimate hazard ratios (HRs) and 95% confidence intervals (CIs) for the associations between quintiles of blood lipid concentrations and aortic aneurysm risk, using time since baseline as the underlying timescale. Analyses using age as the underlying time metric resulted in similar results. Adjustments were made for age, sex, ethnicity (white/non-white), Townsend deprivation index (quintiles), education (none, national exams at age 16 years [O levels/GCSEs or equivalent/CSEs or equivalent], optional national exams at ages 17-18 years [A levels/AS levels or equivalent] or vocational qualifications [NVQ or HND or HNC or equivalent], other professional qualifications [e.g. nursing, teaching], college or university degree, missing), smoking status and cigarettes/day (never, former, current < 20 cigarettes/day, current ≥ 20 cigarettes/day, or current & missing cigarettes/day), alcohol consumption (never, special occasions, 1–2 times/week, 3–4 times/week, 5–7 times/week, missing), BMI categories (< 18.5, 18.5-<25.0, 25.0-<30.0, 30.0-<35.0, ≥ 35.0 kg/m^2^), frequency of physical activity (sum of walking, moderate and vigorous activity) per week (0–6, 7–9, 10–12, 13–15, 16–21 times/week, missing), height (sex-specific quintiles), hypertension (yes/no based on a combination of measured systolic blood pressure ≥ 140 mmHg or diastolic blood pressure ≥ 90 mmHg, combined with self-report of hypertension or hospital diagnosis of hypertension at baseline, ICD-10 code I10 or ICD-9 code 401), and use of lipid-lowering medication. Missing data were in general low (from 0.12% for Townsend deprivation index to 8.32% for physical activity) and were treated as a separate category. Analyses were conducted for aortic aneurysm overall, and by sex, and for abdominal, thoracic, and unspecified aortic aneurysm separately, as well as for ruptured vs. non-ruptured aortic aneurysm. We also analysed the association with aortic aneurysm mortality separately. To take into account reverse causation, we conducted further analyses excluding the first 5 years of follow-up. All statistical tests were two-sided, with *p* < 0.05 considered statistically significant.

### Meta-analysis

#### Search strategy

This systematic review was conducted in accordance with the Preferred Reporting Items for Systematic Reviews and Meta-Analyses (PRISMA) statement [31] and was registered with PROSPERO on 29th July 2022 (CRD42022349849). A search strategy was developed and two databases, PubMed and Embase, were searched up to 11th November 2025, for peer-reviewed studies on blood lipids and the risk of aortic aneurysm (Supplementary text). More specifically, the relevant exposures were total cholesterol, LDL cholesterol, HDL cholesterol, triglycerides, apolipoprotein A1, apolipoprotein B, and lipoprotein(a). The full search strategy is provided in the Supplementary Text. Furthermore, reference lists of included articles were manually searched to identify additional potentially relevant publications.

#### Inclusion and exclusion criteria

The eligibility criteria were determined using the PICO structure. Published peer-reviewed cohort or prospective studies (including case-cohort or nested case-control studies within cohorts) reporting adjusted risk estimates (e.g., odds ratios [ORs], relative risks [RRs], HRs and 95% CIs) for the association between blood lipid concentrations (total cholesterol, LDL cholesterol, HDL cholesterol, triglycerides, apolipoprotein A1, apolipoprotein B, or lipoprotein(a)) and risk of aortic aneurysm were included. English language articles involving adults aged ≥ 18 years old were included. Unrelated studies, duplicated studies, conference abstracts and experimental/laboratory studies and studies on animals were rendered unsuitable. If multiple relevant publications reported on the same lipid within the same study population, only the most comprehensive assessment (largest number of participants and cases) was included.

#### Study selection

Results from both database searches were combined in Reference Manager and title screening was performed and unsuitable records were excluded. The full texts of the remaining records were read and those satisfying all inclusion criteria were incorporated in the review. The screening was done by two authors in duplicate (EI, DA). Discrepancies were resolved by discussion with a third author (AKH).

#### Data extraction

We extracted the following data from each publication: first author name; year of publication; country/location; study name or description; study period; duration of follow-up; number of participants or controls, sex, age, number of cases; outcome(s); blood lipids evaluated as exposures; comparison (the contrast and metric of blood lipids); RR/OR/HR and 95% CI; any adjustments made for covariates. The data extracted was entered in a structured extraction table.

#### Quality assessment

A modified version of the Newcastle-Ottawa Scale (NOS) for assessing the quality of non-randomised studies was used to assess the quality of the studies [32]. This scale scores studies based on participant selection, comparability and ascertainment of the outcome. We modified the original scoring system by (1) removing the point regarding representativeness as this is not a study quality criteria and (2) gave 0.25 point for each confounder adjusted for, up to a maximum of two points, instead of one point for each of two confounders adjusted for, as described previously [33]. The maximum score was therefore reduced from 9 points to 8 points in the modified version of the scale that was used.

### Statistical analysis

A meta-analysis of all relevant published studies was conducted. Each lipid type was modelled individually and analysed if results were available from at least two different studies. For studies that reported total, LDL, non-HDL or HDL cholesterol in mg/dl we converted the concentrations to mmol/L by dividing by 38.67, while for triglycerides we converted from mg/dl to mmol/L by dividing by 88.57 [34]. For conversion of apolipoprotein A1 we used a conversion factor of 28.3, so 1 mmol/L is equal to 1 g/L. Random effects models were used to estimate summary RRs and 95% CIs for the association between blood lipid levels and risk of aortic aneurysm [35]. Linear and non-linear dose-response meta-analyses and high vs. low meta-analyses were conducted and visually presented as forest plots and non-linear dose-response figures. The method of Greenland and Longnecker was used for the linear dose-response analyses [36]. Study-specific linear dose-response slopes were calculated in a logit-linear model using the estimates across at least three categories of lipid levels. This method requires that the distribution of cases and participants or person-years per category is available, but for studies where this information was not available the distributions were estimated based on a method previously described [37]. The mean or median lipid concentration per category was used directly if it was reported in the publications. For studies that reported lipid concentrations in ranges, the average of the lower and upper bounds for each category was used, and for studies reporting open-ended or extreme upper or lower cut-off points for the highest and lowest categories, the width of the adjacent category was used to estimate an upper and lower cut-off value and the corresponding midpoint. If continuous estimates were reported directly in the publication, they were converted (when necessary) to reflect the corresponding increment used for each analysis. Summary RRs were estimated per 1 mmol/L increase for total, LDL and HDL cholesterol and triglycerides, and per 50 mg/dl for lipoprotein(a). Fractional polynomial models were used for the non-linear dose-response analysis [38]. The best fitting second order fractional polynomial regression model, defined as the one with the lowest deviance, was determined. A likelihood ratio test was used to assess the difference between the nonlinear and linear models to test for nonlinearity [38].

The I^2^ test was used to assess heterogeneity between studies [39], with I^2^ values of approximately 25%, 50% and 75% indicating low, moderate and high heterogeneity respectively. Funnel plots were inspected for asymmetry and both Egger’s test [40] and Begg’s test [41] were used to assess publication bias for analyses with at least 7 studies. Potential publication bias was indicated when *p* < 0.10. When there was evidence of possible publication bias, we conducted sensitivity analyses using the Trim and Fill method to assess the potential impact of publication bias on the summary estimates [42]. Sensitivity analyses excluding one study at a time from each analysis was conducted to assess the impact of each study on the overall results and was done for dose-response analyses with at least 5 studies included. All statistical analyses were done using Stata version 16.1 (Stata-Corp, College Station, Texas, USA).

## Results

### UK Biobank

The analytical dataset included 429,610 participants, and after an average of 12.3 years follow-up (5,297,095 person-years for aortic aneurysm incidence and 5,306,264 person-years for aortic aneurysm mortality), a total of 2434 incident aortic aneurysm cases (71 of these were from death records only) and 122 aortic aneurysm deaths occurred. Participants with higher total cholesterol tended to be older, and more women and white participants had higher total cholesterol (Table 1). In addition, those with higher cholesterol were on average less deprived, had lower education, were more likely to be hypertensive and less likely to use lipid-lowering medications, but little differences were observed in smoking status, BMI, physical activity and height across quintiles of total cholesterol (Table 1). Characteristics of study participants across quintiles of LDL, non-HDL cholesterol, HDL cholesterol, triglycerides and lipoprotein(a) are shown in Supplementary Tables 1–5.

**Table 1:** Characteristics of participants in the UK biobank study by quintiles of total cholesterol concentrations

		Total cholesterol, mmol/L (median)				
		Quintile 1	Quintile 2	Quintile 3	Quintile 4	Quintile 5
		4.393	5.158	5.714	6.295	7.196
Participants (n)		85,983	86,004	85,822	85,916	85,885
Age, years (median)		56	56	57	58	59
Sex (%)	Men	54.0	47.1	44.5	41.7	34.2
	Women	46.0	52.9	55.5	58.9	65.8
Ethnicity (%)	White	91.9	93.9	94.7	95.6	96.4
	Non-white	7.7	5.8	5.0	4.1	3.2
	Missing	0.4	0.3	0.3	0.3	0.3
Townsend Deprivation Index (highest) (%)		22.3	19.7	18.5	17.7	17.0
Education (highest) (%)		33.6	34.8	33.8	33.1	31.5
Smoking status (%)	Never	55.0	56.7	56.5	56.0	55.1
	Former	34.1	33.2	33.3	34.0	34.5
	Current	10.9	10.1	10.2	10.0	10.4
BMI (median)		26.9	26.4	26.4	26.6	26.8
Physical activity, frequency/week (median)		11.0	11.0	11.0	11.0	11.0
Height, cm, men/women (median)		176.0/163.0	176.0/163.0	176.0/163.0	176.0/162.0	175.0/162.0
Hypertension (%)		53.8	49.6	51.0	53.7	57.7
Use of lipid-lowering medications (%)		36.1	15.6	8.5	4.7	3.4

In the multivariable model, after adjustment for age, sex, ethnicity, Townsend Deprivation Index, education, smoking status and cigarettes per day, BMI, physical activity, height, hypertension, and lipid-lowering medications, we observed positive associations between the highest vs. lowest quintile of total cholesterol (HR 1.22, 95% CI 1.07–1.41), LDL cholesterol (1.39, 1.21–1.60), apolipoprotein B (1.52, 1.33–1.74), non-HDL cholesterol (1.50, 1.29–1.73), triglycerides (1.23, 1.06–1.42), lipoprotein(a) (1.34, 1.17–1.54) and aortic aneurysm risk, while HDL cholesterol (0.57, 0.48–0.67) and apolipoprotein A1 (0.54, 0.46–0.63) were inversely associated (Table 2). These associations were strengthened for total cholesterol (1.30, 1.12–1.53), LDL cholesterol (1.46, 1.25–1.71), apolipoprotein B (1.57, 1.35–1.83), and non-HDL cholesterol (1.58, 1.34–1.86) (Supplementary Tables 6–9), but remained similar for the remaining lipids (Supplementary Tables 10-13), when the first 5 years of follow-up was excluded. The positive associations for total cholesterol, LDL cholesterol, apolipoprotein B, non-HDL cholesterol, triglycerides, and lipoprotein(a) tended to be stronger in men than women. The positive and inverse associations tended to be most pronounced for abdominal aortic aneurysm, and null or weaker for thoracic aortic aneurysm and unspecified aortic aneurysm (Supplementary Tables 6–13). When stratified by whether the aneurysm ruptured or not, associations were in general similar to the main analysis for non-ruptured aortic aneurysm, and statistically non-significant for ruptured aortic aneurysm, however, there were few cases of ruptured aortic aneurysm (Supplementary Tables 6–13).

**Table 2:** Associations between quintiles of blood lipid concentration and aortic aneurysm in UK biobank

	Total cholesterol, mmol/L (median)					*p*_trend_	Continuous
	Quintile 1	Quintile 2	Quintile 3	Quintile 4	Quintile 5		
	4.393	5.158	5.714	6.295	7.196		Per 1 mmol/L
Participants	85,983	86,004	85,822	85,916	85,885		429,610
Cases	615	492	448	440	439		2,434
HR (95% CI)	1.00	1.12 (0.99–1.27)	1.10 (0.97–1.26)	1.14 (0.99–1.30)	1.22 (1.07–1.41)	0.007	1.05 (1.01–1.09)
	LDL cholesterol, mmol/L (median)					p_trend_	Continuous
	2.558	3.135	3.567	4.020	4.713		Per 1 mmol/L
Participants	86,000	85,927	85,979	85,907	85,797		429,610
Cases	551	457	463	475	488		2,434
HR (95% CI)	1.00	1.14 (1.00-1.30)	1.25 (1.09–1.43)	1.30 (1.13–1.49)	1.39 (1.21–1.60)	< 0.001	1.14 (1.08–1.20)
	Apolipoprotein B, g/L (median)					p_trend_	Continuous
	0.756	0.910	1.029	1.154	1.350		Per 1 g/L
Participants	86,456	86,047	85,954	85,364	85,789		429,610
Cases	470	449	488	458	569		2434
HR (95% CI)	1.00	1.14 (1.00-1.30)	1.28 (1.12–1.46)	1.23 (1.07–1.42)	1.52 (1.33–1.74)	< 0.001	1.76 (1.47–2.10)
	Non-HDL cholesterol, mmol/L (median)					p_trend_	Continuous
	3.002	3.702	4.237	4.804	5.678		Per 1 mmol/L
Participants	78,712	78,763	78,581	78,615	78,741		393,412
Cases	454	421	427	439	491		2,232
HR (95% CI)	1.00	1.18 (1.03–1.35)	1.28 (1.11–1.47)	1.31 (1.13–1.51)	1.50 (1.29–1.73)	< 0.001	1.13 (1.08–1.18)
	HDL cholesterol, mmol/L (median)						Continuous
	1.020	1.232	1.413	1.624	1.972		Per 1 mmol/L
Participants	78,878	78,515	78,871	78,635	78,513		393,412
Cases	810	550	374	288	210		2,232
HR (95% CI)	1.00	0.84 (0.75–0.94)	0.68 (0.60–0.77)	0.65 (0.56–0.74)	0.57 (0.48–0.67)	< 0.001	0.55 (0.48–0.64)
	Apolipoprotein A1, g/L (median)					p_trend_	Continuous
	1.226	1.391	1.520	1.663	1.910		Per 1 g/L
Participants	78,581	78,087	78,284	78,262	77,879		391,093
Cases	751	561	358	324	228		2,222
HR (95% CI)	1.00	0.86 (0.77–0.96)	0.62 (0.55–0.71)	0.65 (0.57–0.75)	0.54 (0.46–0.63)	< 0.001	0.39 (0.32–0.47)
	Triglycerides, mmol/L (median)					p_trend_	Continuous
	0.787	1.118	1.471	1.956	2.981		Per 1 mmol/L
Participants	86,174	85,821	85,829	85,889	85,897		429,610
Cases	275	428	474	558	699		2,434
HR (95% CI)	1.00	1.18 (1.01–1.37)	1.09 (0.94–1.27)	1.11 (0.96–1.29)	1.23 (1.06–1.42)	0.022	1.05 (1.01–1.09)
	Lipoprotein(a), mg/dl (median)					p_trend_	Continuous
	5.70	11.06	20.90	48.79	130.40		Per 50 mg/dl
Participants	69,028	68,942	69,055	68,941	68,992		344,958
Cases	362	366	357	395	450		1,930
HR (95% CI)	1.00	1.04 (0.90–1.20)	1.02 (0.88–1.19)	1.19 (1.03–1.37)	1.34 (1.17–1.54)	< 0.001	1.27 (1.16–1.39)

When aortic aneurysm mortality was examined, the results were mixed with null associations for total cholesterol (HR for highest vs. lowest quintile 0.68, 95% CI 0.34–1.34), LDL cholesterol (0.92, 0.47–1.80), apolipoprotein B (1.34, 0.69–2.58), non-HDL cholesterol (0.89, 0.44–1.80) and triglycerides (1.07, 0.55–2.09), but a positive associations was observed for lipoprotein(a) (2.27, 1.22–4.20), while HDL cholesterol (0.36, 0.15–0.85) and apolipoprotein A1 (0.42, 0.20–0.88) were inversely associated (Supplementary Tables 6–13).

### Meta-analysis

#### Literature search

**Fig. 1:**
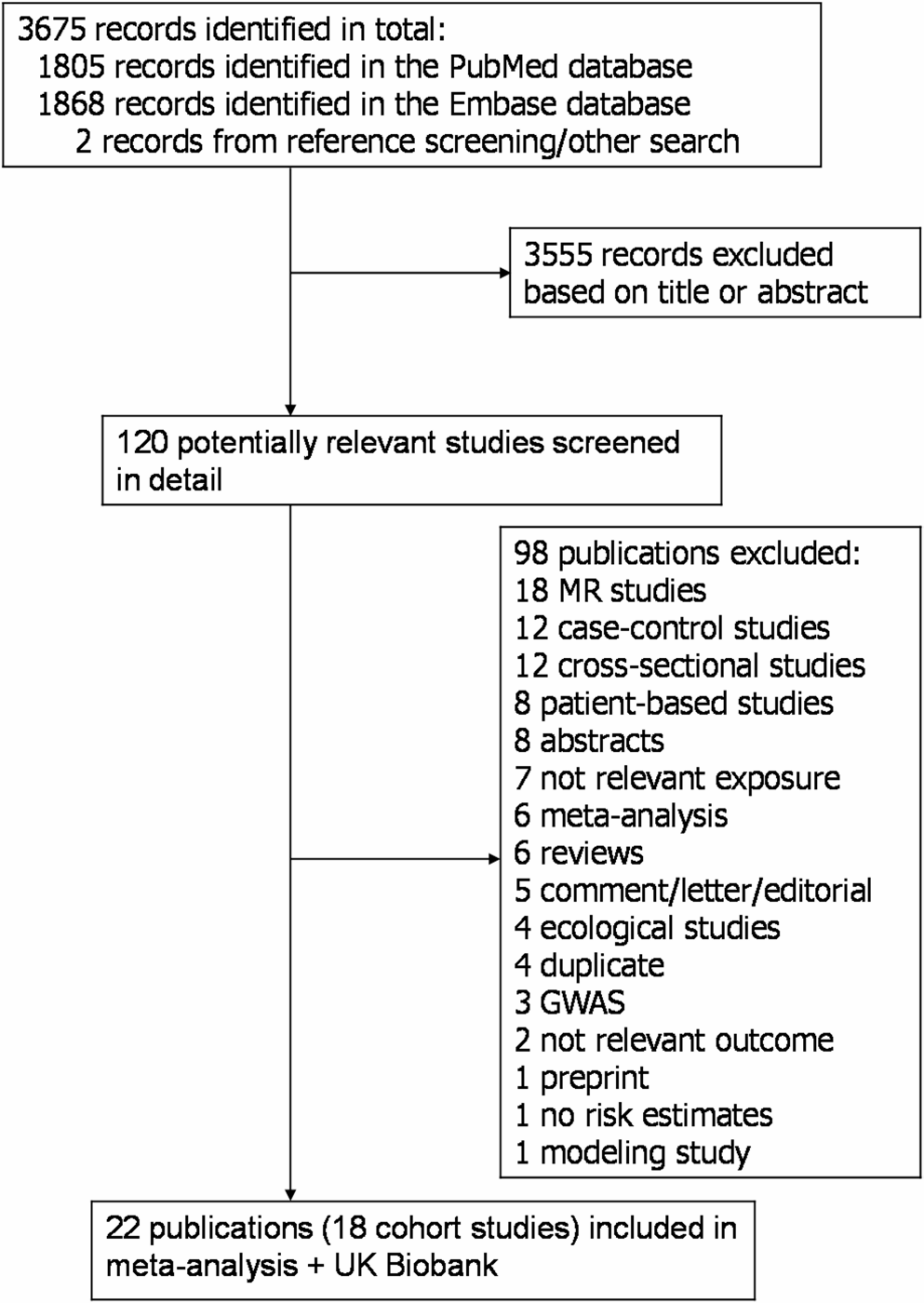
Flow-chart of study selection for systematic review and meta-analysis of blood lipids and aortic aneurysm risk

The database searches generated a total of 3,675 records, and 3,555 records were excluded based on screening of title and abstract, leaving 120 records for full-text review. Out of these, 98 records were excluded due to various reasons (Fig. 1, Supplementary Table 14), leaving 22 publications with data from 18 cohort studies included in the systematic review and meta-analysis [3, 5, 8, 18–28, 43–50] in addition to the UK Biobank study (Supplementary Table 15). Two publications reported results from the same cohort [8, 20], but one reported categorical results (included in the nonlinear dose-response analysis) [20], and the second reported continuous results (included in the linear dose-response analysis) [8].

#### Study characteristics

The characteristics of the studies included are summarized in Supplementary Table 15. Twelve studies were from Europe (including the present study), five studies were from the USA and two studies were from Asia. Five studies were in men only, while thirteen included both men and women, and one included women only. Fourteen studies had a follow-up time ≥ 10 years, while only five studies had a follow-up duration of < 10 years. The number of participants varied from 175 in a nested case-control study to 4,162,640 in a Korean study, and the number of cases ranged from 35 to 18,160. Four studies reported on aortic aneurysm overall and fifteen studies reported on abdominal aortic aneurysm, and only two reported on thoracic aortic aneurysm.

#### Study quality assessment

The results of the NOS study quality assessment process are presented in Supplementary Table 16. Study scores were excellent, ranging from 5.25 to 8 across publications, with an average score of 6.88 and a median score of 7 out of a maximum of 8, suggesting the studies were of generally good quality.

### Meta-analysis

#### Total cholesterol

**Fig. 2:**
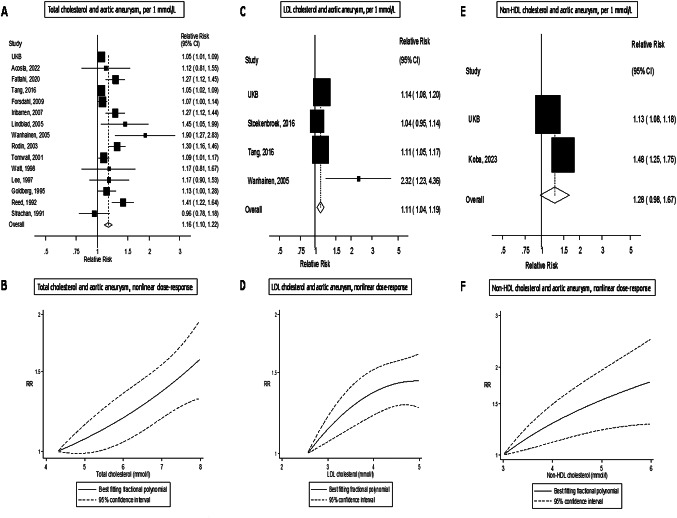
Linear and nonlinear dose-response meta-analysis of total, LDL and non-HDL cholesterol and aortic aneurysm

A total of 15 prospective studies (14 published studies [3, 8, 18–25, 27, 43–45] and UK Biobank) with 5,219 cases and 635,267 participants were included in the meta-analysis of total cholesterol and the risk of aortic aneurysm. In the linear dose-response analysis, the summary RR per 1 mmol/L increment in total cholesterol was 1.16 (95% CI 1.10–1.22, I^2^ = 74%, p_heterogeneity_<0.001) (Fig. 2a). The summary RR ranged from 1.14 (1.08–1.19) when excluding the study by Reed et al. [8], to 1.18 (1.11–1.26) when excluding the study by Tang et al. [3] (Supplementary Fig. 1). There was indication of publication bias with Egger’s test (*p* = 0.003), and with Begg’s test (*p* = 0.09) (Supplementary Fig. 2). When using the trim and fill method, seven studies were added to the plot and the summary RR became 1.07 (1.01–1.13). Seven studies (six published studies [3, 19–21, 44, 45] and UK Biobank) were included in the high vs. low analysis (4,163 cases, 578,103 participants), the summary RR was 1.75, 1.33–2.30, I^2^ = 67%, *n* = 7) (Supplementary Fig. 3). There was no indication of nonlinearity in the nonlinear dose-response analysis and there was a positive dose-response relationship (p_nonlinearity_=0.18; Fig. 2b).

#### LDL cholesterol

A total of four studies (three published studies [3, 27, 46] and UK Biobank) with 3,277 cases and 457,494 participants were included in the analysis of LDL cholesterol and aortic aneurysm. The summary RR per 1 mmol/L increase in LDL cholesterol was 1.11 (1.04–1.19, I^2^ = 63%) (Fig. 2c). Three studies (two published studies [3, 46] and UK Biobank) were included in the high vs. low analysis (3,242 cases, 457,319 participants) and the summary RR was 1.43 (1.27–1.60, I^2^ = 0%) (Supplementary Fig. 4). The test for nonlinearity was not significant (p_nonlinearity_=0.09) and there was evidence of a positive dose-response relationship between increasing LDL cholesterol and aortic aneurysm risk (Fig. 2d).

#### Apolipoprotein B

Three studies (two published studies [44, 47] and UK Biobank) were included in a high vs. low analysis of apolipoprotein B and aortic aneurysm (2,636 cases, 460,202 participants). The summary RR was 1.72 (1.24–2.38, I^2^ = 60%) (Supplementary Fig. 5).

#### Non-HDL cholesterol

Two studies (one published study [26] and UK Biobank) were included in the meta-analysis of non-HDL cholesterol and aortic aneurysm (2,624 cases, 525,333 participants) and the summary RR per 1 mmol/L was 1.28 (0.98–1.67, I^2^ = 89%) (Fig. 2e) and for high vs. low levels was 1.70 (1.20–2.40, I^2^ = 60%) (Supplementary Fig. 6). There was a positive dose-response relationship and no evidence for a nonlinear association (p_nonlinearity_=0.28) (Fig. 2f).

#### HDL cholesterol

**Fig. 3:**
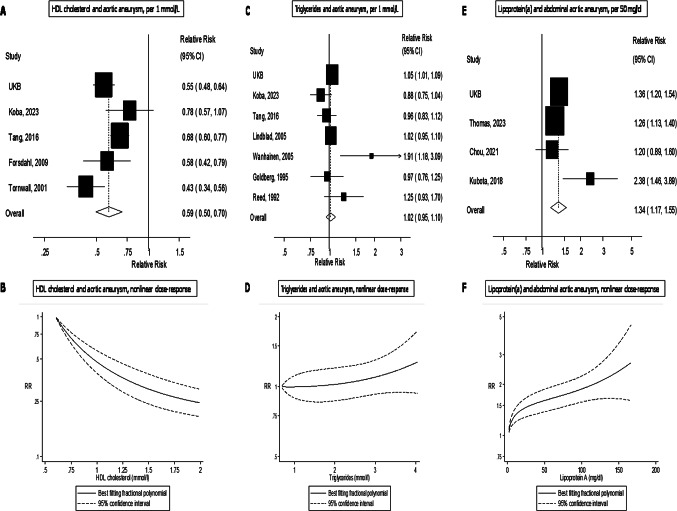
Linear and nonlinear dose-response meta-analysis of HDL cholesterol, triglycerides and lipoprotein(a) and aortic aneurysm

Five studies (four published studies [3, 19, 21, 26] and UK Biobank) with 3,387 cases among 528,513 participants were included in the meta-analysis of HDL cholesterol levels and the risk of aortic aneurysm. In the linear dose-response analysis, the summary RR per 1 mmol/L increment in HDL cholesterol was 0.59 (0.50–0.70, I^2^ = 72%, p_heterogeneity_<0.001) (Fig. 3a). The summary RR ranged from 0.56 (0.46–0.69) when excluding the study by Tang et al. [3] to 0.63 (0.54–0.73) when excluding the study by Tornwall et al. [21] (Supplementary Fig. 7). Six studies (five published studies [3, 19, 21, 26, 49] and UK Biobank) were included in the high vs. low analysis (21,547 cases, 4,691,153 participants) and the summary RR was 0.47 (0.36–0.61, I^2^ = 86%) (Supplementary Fig. 8). There was strong evidence of nonlinearity (p_nonlinearity_<0.001) with a steeper reduction in risk at lower HDL cholesterol levels than at higher levels (Fig. 3b).

#### Apolipoprotein A1

Two studies (one published [44] and UK Biobank) were included in the dose-response analysis of apolipoprotein A1 and aortic aneurysm (2,252 cases, 391,273 participants and the summary RR was 0.59 (0.26–1.35, I^2^ = 98%) (Supplementary Fig. 9). Three studies (two published studies [44, 47] and UK Biobank) were included in a high vs. low analysis of apolipoprotein A1 and aortic aneurysm (2,424 cases, 421,685 participants). The summary RR was 0.52 (0.40–0.68, I^2^ = 45%) (Supplementary Fig. 10).

#### Triglycerides

Seven studies (six published studies [3, 8, 18, 20, 26, 27] and UK Biobank) with 3,720 cases and 542,063 participants were included in the analysis of triglycerides and aortic aneurysm. The summary RR per 1 mmol/L increase in triglycerides was 1.02 (0.95–1.10, I^2^ = 55%, p_heterogeneity_=0.06) (Fig. 3c). The summary RR ranged from 1.01 (0.94–1.09) when excluding the study by Reed et al. [8], to 1.04 (0.97–1.12) when excluding the study by Koba et al. [26] (Supplementary Fig. 11). There was no indication of publication bias with Egger’s test (*p* = 0.87) or Begg’s test (*p* = 0.55) (Supplementary Fig. 12). Six studies (five published [3, 20, 26, 44, 49] and UK Biobank) were included in a high vs. low analysis of triglycerides and aortic aneurysm (21,598 cases, 4,696,774 participants) and the summary RR was 1.16 (0.93–1.45, I^2^ = 81%) (Supplementary Fig. 13). The test for nonlinearity was not statistically significant (p_nonlinearity_=0.42), and there was no clear association between triglycerides and aortic aneurysm across the observed range of triglyceride concentrations (Fig. 3d).

#### Lipoprotein(a)

Four studies (three published studies [5, 28, 50] and UK Biobank) with 3,502 cases and 435,573 participants were included in the analysis of lipoprotein(a) and abdominal aortic aneurysm. The summary RR per 50 mg/dl increase in lipoprotein A was 1.34 (1.17–1.55, I^2^ = 57%, p_heterogeneity_=0.07) (Fig. 3e). Five studies (four published studies [5, 28, 44, 50] and UK Biobank) were included in the high vs. low analysis (3,532 cases, 435,753 participants) and the summary RR was 1.40 (1.25–1.57, I^2^ = 0%) (Supplementary Fig. 14). The test for nonlinearity was significant (p_nonlinearity_=0.01) and the steepest increase in risk was observed at the low end of the curve and there was a positive dose-response relationship between increasing levels of lipoprotein(a) and aortic aneurysm risk (Fig. 3f).

## Discussion

The findings from the UK Biobank suggest higher blood concentrations of total cholesterol, LDL cholesterol, apolipoprotein B, non-HDL cholesterol, triglycerides, and lipoprotein(a) are associated with a 22–52% increased risk of aortic aneurysm when comparing the highest versus lowest quintile, while higher concentrations of HDL cholesterol and apolipoprotein A1 were associated with a 43–46% lower risk of aortic aneurysm. These associations were in general more apparent for abdominal aortic aneurysms than for thoracic and unspecified aortic aneurysms, and for non-ruptured than for ruptured aortic aneurysms. The results for total cholesterol, LDL cholesterol, apolipoprotein B, non-HDL cholesterol, lipoprotein(a), HDL cholesterol, and apolipoprotein A1 were further confirmed in a meta-analysis of cohort studies, however, the association for triglycerides was null in the meta-analysis. While there was a moderate to large number of studies on total cholesterol and triglycerides, the number of studies on the other circulating lipids was limited, and in some cases only allowed for high vs. low meta-analyses due to the way the results were reported (e.g. only dichotomous results, or no quantification of the lipid level).

The current findings are consistent with several Mendelian randomization studies which reported increased risk of aortic aneurysm with higher total and LDL cholesterol [51–54], and lipoprotein(a) [50, 55], and reduced risk with higher HDL cholesterol [51, 54], providing some support for potential causal relationships. In addition, cohort studies have shown cholesterol-lowering medications, such as statins, to be associated with lower risk of ruptured aortic aneurysm [56, 57], and reduced growth rate of aortic aneurysms [56], providing some further evidence in support of causality. As for the biological mechanism that may explain the observed associations, there is evidence that atherosclerotic changes of the aortic wall may play a part in the pathogenesis of aortic aneurysms [8, 58]. Aortic aneurysms are characterized by elastin degradation, which may be mediated by inflammation [59]. Chronic inflammation with infiltration of the outer wall by lymphocytes and macrophages is observed in aortic aneurysms [59, 60]. Lipoprotein(a) is a main carrier of proinflammatory oxidized phospholipids [61], which can contribute to aortic aneurysm development by activating coagulation in abdominal aortic aneurysm lesions [62]. Oxidized LDL cholesterol can initiate endothelial and smooth muscle cell damage [63, 64], induce production of platelet derived growth factors [65], and stimulate the migration of smooth muscle cells from media to intima [66]. Oxidized LDL cholesterol is taken up to a greater extent by monocyte-derived macrophages than LDL cholesterol [67], and the uptake induces an inflammatory response resulting in the release of matrix metalloproteinases and urokinase-type plasminogen activator which contribute to the extracellular matrix remodelling [68] and play a major role in aortic aneurysm development and progression [69].

Strengths of the UK Biobank analysis include the large sample size and relatively long follow-up with a large number of incident aortic aneurysm cases, comprehensive assessment and adjustment for confounding factors, and detailed analyses of aortic aneurysm subtypes. Strengths of the meta-analysis include the prospective design of the included studies, the large number of included studies, participants, and cases, and detailed dose-response analyses. The analyses also have some limitations. A limitation of the UK Biobank study is that the participation rate was only 5.5%, thus there may be healthy volunteer effects in the study. However, causal risk factors for diseases can still be detected even if the sample is not fully representative of the general population. In addition, the results from the UK Biobank were in general consistent with most of the remaining studies included in the meta-analysis, alleviating some concern regarding this point. Since aortic aneurysm diagnoses were based on a combination of self-report, hospital records and death records, and there was no screening for aortic aneurysm at the baseline visit, it is possible that some of the incident aortic aneurysm cases may already have existed, but been undiagnosed, at baseline. However, such initially undiagnosed cases would most likely have been diagnosed in the early part of the follow-up period and in analyses excluding the first 5 years of follow-up, the results persisted and in some cases were slightly strengthened (e.g. total cholesterol, LDL cholesterol, non-HDL cholesterol, and apolipoprotein B), thus alleviating any cause for concern in this regard. Since we used regular Cox regression analyses it is possible that we may have overestimated aortic aneurysm risk somewhat due to competing risks. Most studies only had a baseline assessment of lipids and were not able to take into account changes in lipids over time, however, given the prospective design of the included studies it is likely that this would have resulted in bias toward the null. Most of the studies were conducted in Europe and the USA, and only two studies were from Asia. Nevertheless, the results across the studies were directionally consistent across regions as well as in one US study among Japanese men suggesting the findings may be generalisable across these regions and ethnicities. However, additional studies would be useful to further assess this. As with any meta-analysis, publication bias is a possibility. We found some evidence of possible publication bias in the analysis of total cholesterol and aortic aneurysm, but not for triglycerides. When using the trim and fill method, the association was attenuated, but remained statistically significant and positive, however, we cannot be sure whether the asymmetry in the funnel plot really was due to publication bias or due to other study characteristics correlated with study size. For the remaining exposures, too few studies were included for publication bias tests to be done, thus further studies are warranted. There was high heterogeneity in some of the analyses, particularly for total, LDL, and HDL cholesterol, however, this appeared to be more driven by differences in the magnitude of the associations rather than differences in the direction of the associations as all the studies were directionally consistent.

The current findings have important public health implications. Healthy dietary and lifestyle changes that can reduce total and LDL cholesterol levels could contribute to lower risk of aortic aneurysm and several other diseases [53]. In addition to elevated lipids being associated with increased aortic aneurysm risk, previous meta-analyses have confirmed strong associations between smoking [12, 13] and hypertension [14] and increased aortic aneurysm risk, and suggested a possible reduced risk with high physical activity [15]. This suggests adoption of healthy lifestyle behaviours and improvements in several risk factors that are known to be beneficial for a range of other diseases [70–72], is also likely to be beneficial in reducing risk of aortic aneurysm. Any additional studies could further assess the associations between blood lipids and aortic aneurysm subtypes and mortality. In addition, further studies from other regions than Europe and the USA would be informative.

In conclusion, this analysis of the UK Biobank found positive associations between total cholesterol, LDL cholesterol, apolipoprotein B, triglycerides, and lipoprotein(a) and aortic aneurysm risk, and inverse associations for HDL cholesterol and apolipoprotein A1. These associations were in general most pronounced for abdominal aortic aneurysm, and less pronounced for other subsites. Results for total cholesterol, LDL cholesterol, HDL cholesterol and lipoprotein(a) were further confirmed in a meta-analysis of cohort studies, but the association with triglycerides was null. These findings suggest blood lipid levels may be important risk factors for aortic aneurysm, however, further studies are needed across aortic aneurysm subsites.

## Supplementary Information

Below is the link to the electronic supplementary material.

Supplementary Material 1
