# Supplementary material for: Blood lipids and the risk of aortic aneurysm: results from the UK Biobank study and a systematic review and meta-analysis of cohort studies
Source: Eur J Epidemiol. 2026 Jan 24;41(4):405–17. doi: 10.1007/s10654-025-01344-4 (PMC13331847; doi:10.1007/s10654-025-01344-4)
Supplement: Supplementary file 1 — Supplementary Material 1 [file 10654_2025_1344_MOESM1_ESM.docx]

**Supplemental material for**

Ioannidou E, Aune D, Theodosopoulos I, Heath AK. Blood lipids and the risk of aortic aneurysm: results from the UK Biobank study and a systematic review and meta-analysis of cohort studies.

**Supplementary Table 1. Characteristics of participants in the UK Biobank study by quintiles of LDL cholesterol concentrations**

|  | | | LDL cholesterol, mmol/L (median) | | | | | | |
| --- | --- | --- | --- | --- | --- | --- | --- | --- | --- |
|  | | | Quintile 1 | Quintile 2 | | Quintile 3 | | Quintile 4 | Quintile 5 |
|  | | | 2.558 | 3.135 | | 3.567 | | 4.020 | 4.713 |
| Participants (n) | | | 86,000 | 85,927 | | 85,979 | | 85,907 | 85,797 |
| Age, years (median) | | | 56 | 56 | | 57 | | 58 | 59 |
| Sex (%) | Men | | 47.0 | 44.1 | | 44.9 | | 44.7 | 40.2 |
|  | Women | | 53.0 | 55.9 | | 55.1 | | 55.3 | 59.8 |
| Ethnicity (%) | White | | 92.5 | 94.0 | | 94.6 | | 95.3 | 96.1 |
|  | Non-white | | 7.1 | 5.7 | | 5.1 | | 4.4 | 3.5 |
|  | Missing | | 0.4 | 0.3 | | 0.3 | | 0.3 | 0.3 |
| Townsend Deprivation Index (highest) | | | 21.9 | 19.4 | | 18.4 | | 18.0 | 17.5 |
| Education (highest) | | | 34.1 | 34.8 | | 33.8 | | 33.2 | 30.9 |
| Smoking status (%) | | Never | 54.6 | | 56.6 | | 56.9 | 56.2 | 55.0 |
|  |  | Former | 35.0 | | 33.4 | | 33.3 | 33.6 | 33.9 |
|  |  | Current | 10.5 | | 10.0 | | 9.8 | 10.2 | 11.1 |
| BMI (median) | | | 26.3 | 26.2 | | 26.5 | | 26.8 | 27.2 |
| Physical activity, frequency/week (median) | | | 11.0 | 11.0 | | 11.0 | | 11.0 | 11.0 |
| Height, cm, men/women (median) | | | 175.5/163.0 | 176.0/163.0 | | 176.0/163.0 | | 176.0/162.0 | 175.5/162.0 |
| Hypertension (%) | | | 53.0 | 49.8 | | 51.3 | | 54.1 | 57.6 |
| Use of lipid-lowering medications (%) | | | 37.2 | 15.8 | | 7.9 | | 4.2 | 3.2 |

**Supplementary Table 2. Characteristics of participants in the UK Biobank study by quintiles of non-HDL cholesterol concentrations**

|  | | | Non-HDL cholesterol, mmol/L (median) | | | | | | |
| --- | --- | --- | --- | --- | --- | --- | --- | --- | --- |
|  | | | Quintile 1 | Quintile 2 | | Quintile 3 | | Quintile 4 | Quintile 5 |
|  | | | 3.002 | 3.702 | | 4.237 | | 4.804 | 5.678 |
| Participants (n) | | | 78,712 | 78,763 | | 78,581 | | 78,615 | 78,741 |
| Age, years (median) | | | 56 | 56 | | 57 | | 58 | 58 |
| Sex (%) | Men | | 44.2 | 43.5 | | 44.9 | | 46.5 | 44.0 |
|  | Women | | 55.8 | 56.5 | | 55.1 | | 53.5 | 56.0 |
| Ethnicity (%) | White | | 92.5 | 94.0 | | 94.6 | | 95.2 | 96.1 |
|  | Non-white | | 7.2 | 5.6 | | 5.1 | | 4.5 | 3.6 |
|  | Missing | | 0.3 | 0.4 | | 0.3 | | 0.3 | 0.3 |
| Townsend Deprivation Index (highest) | | | 21.6 | 19.4 | | 18.5 | | 18.0 | 17.9 |
| Education (highest) | | | 35.2 | 34.6 | | 33.6 | | 32.7 | 30.3 |
| Smoking status (%) | | Never | 55.6 | | 56.6 | | 56.9 | 55.8 | 54.1 |
|  |  | Former | 34.4 | | 33.6 | | 33.3 | 33.8 | 34.1 |
|  |  | Current | 10.0 | | 9.8 | | 9.8 | 10.4 | 11.8 |
| BMI (median) | | | 25.9 | 26.1 | | 26.5 | | 27.0 | 27.5 |
| Physical activity, frequency/week (median) | | | 11.0 | 11.0 | | 11.0 | | 11.0 | 11.0 |
| Height, cm, men/women (median) | | | 176.0/163.0 | 176.0/163.0 | | 176.0/163.0 | | 176.0/162.0 | 175.5/162.0 |
| Hypertension (%) | | | 50.7 | 49.8 | | 51.9 | | 55.1 | 59.2 |
| Use of lipid-lowering medications (%) | | | 34.2 | 16.6 | | 9.1 | | 5.1 | 3.7 |

**Supplementary Table 3. Characteristics of participants in the UK Biobank study by quintiles of HDL cholesterol concentrations**

|  | | | HDL cholesterol, mmol/L (median) | | | | | | |
| --- | --- | --- | --- | --- | --- | --- | --- | --- | --- |
|  | | | Quintile 1 | Quintile 2 | | Quintile 3 | | Quintile 4 | Quintile 5 |
|  | | | 1.020 | 1.232 | | 1.413 | | 1.624 | 1.972 |
| Participants (n) | | | 78,878 | 78,515 | | 78,871 | | 78,635 | 78,513 |
| Age, years (median) | | | 56 | 57 | | 57 | | 57 | 58 |
| Sex (%) | Men | | 75.2 | 57.9 | | 43.4 | | 29.6 | 83.1 |
|  | Women | | 24.8 | 42.1 | | 56.6 | | 70.4 | 16.9 |
| Ethnicity (%) | White | | 92.6 | 93.9 | | 94.7 | | 95.3 | 96.0 |
|  | Non-white | | 7.0 | 5.8 | | 5.0 | | 4.4 | 3.7 |
|  | Missing | | 0.4 | 0.4 | | 0.3 | | 0.3 | 0.3 |
| Townsend Deprivation Index (highest) | | | 22.6 | 19.9 | | 18.4 | | 17.7 | 16.6 |
| Education (highest) | | | 30.2 | 31.7 | | 33.0 | | 34.5 | 36.8 |
| Smoking status (%) | | Never | 51.9 | | 55.0 | | 56.4 | 58.4 | 57.2 |
|  |  | Former | 33.6 | | 33.5 | | 33.7 | 33.0 | 35.4 |
|  |  | Current | 14.5 | | 11.5 | | 9.9 | 8.6 | 17.4 |
| BMI (median) | | | 28.8 | 26.5 | | 26.7 | | 25.6 | 24.4 |
| Physical activity, frequency/week (median) | | | 10.0 | 10.0 | | 11.0 | | 11.0 | 12.0 |
| Height, cm, men/women (median) | | | 176.0/162.0 | 176.0/162.0 | | 176.0/162.0 | | 176.0/163.0 | 175.0/163.0 |
| Hypertension (%) | | | 60.2 | 56.4 | | 53.0 | | 49.1 | 47.9 |
| Use of lipid-lowering medications (%) | | | 21.4 | 16.1 | | 12.7 | | 10.1 | 8.2 |

**Supplementary Table 4. Characteristics of participants in the UK Biobank study by quintiles of triglyceride concentrations**

|  | | | Triglycerides, mmol/L (median) | | | | | | |
| --- | --- | --- | --- | --- | --- | --- | --- | --- | --- |
|  | | | Quintile 1 | Quintile 2 | | Quintile 3 | | Quintile 4 | Quintile 5 |
|  | | | 0.787 | 1.118 | | 1.470 | | 1.956 | 2.981 |
| Participants (n) | | | 86,174 | 85,821 | | 85,829 | | 85,889 | 85,897 |
| Age, years (median) | | | 54 | 57 | | 58 | | 59 | 58 |
| Sex (%) | Men | | 30.7 | 36.9 | | 42.7 | | 49.7 | 61.0 |
|  | Women | | 69.3 | 63.1 | | 57.3 | | 50.3 | 39.0 |
| Ethnicity (%) | White | | 92.8 | 94.4 | | 95.0 | | 95.2 | 95.1 |
|  | Non-white | | 6.8 | 5.3 | | 4.6 | | 4.5 | 4.6 |
|  | Missing | | 0.3 | 0.3 | | 0.4 | | 0.3 | 0.3 |
| Townsend Deprivation Index (highest) | | | 19.3 | 18.4 | | 18.4 | | 18.8 | 20.3 |
| Education (highest) | | | 39.7 | 35.0 | | 32.6 | | 30.6 | 28.9 |
| Smoking status (%) | | Never | 60.9 | | 58.4 | | 56.4 | 53.7 | 49.9 |
|  |  | Former | 31.0 | | 32.5 | | 33.8 | 35.3 | 36.6 |
|  |  | Current | 8.1 | | 9.1 | | 9.9 | 11.1 | 13.5 |
| BMI (median) | | | 24.4 | 25.6 | | 26.7 | | 27.7 | 28.7 |
| Physical activity, frequency/week (median) | | | 11.0 | 11.0 | | 11.0 | | 10.0 | 10.0 |
| Height, cm, men/women (median) | | | 176.0/164.0 | 176.0/163.0 | | 176.0/162.0 | | 176.0/162.0 | 176.0/162.0 |
| Hypertension (%) | | | 38.5 | 48.1 | | 54.5 | | 59.9 | 64.9 |
| Use of lipid-lowering medications (%) | | | 8.5 | 11.3 | | 13.7 | | 16.1 | 18.8 |

**Supplementary Table 5. Characteristics of participants in the UK Biobank study by quintiles of lipoprotein(a) concentrations**

|  | | | Lipoprotein(a), mg/dl (median) | | | | | | |
| --- | --- | --- | --- | --- | --- | --- | --- | --- | --- |
|  | | | Quintile 1 | Quintile 2 | | Quintile 3 | | Quintile 4 | Quintile 5 |
|  | | | 4.372 | 6.830 | | 11.344 | | 24.133 | 61.573 |
| Participants (n) | | | 69,028 | 68,942 | | 69,055 | | 68,941 | 68,992 |
| Age, years (median) | | | 57 | 57 | | 58 | | 58 | 57 |
| Sex (%) | Men | | 46.9 | 46.0 | | 42.9 | | 40.8 | 44.0 |
|  | Women | | 53.1 | 54.0 | | 57.1 | | 59.2 | 56.0 |
| Ethnicity (%) | White | | 96.8 | 96.0 | | 94.7 | | 90.2 | 93.0 |
|  | Non-white | | 2.9 | 3.7 | | 5.0 | | 9.4 | 6.7 |
|  | Missing | | 0.3 | 0.3 | | 0.3 | | 0.4 | 0.3 |
| Townsend Deprivation Index (highest) | | | 18.3 | 18.3 | | 18.3 | | 20.8 | 19.7 |
| Education (highest) | | | 34.0 | 34.3 | | 33.1 | | 33.0 | 33.5 |
| Smoking status (%) | | Never | 55.4 | | 55.8 | | 55.8 | 56.5 | 56.4 |
|  |  | Former | 34.2 | | 33.8 | | 34.0 | 33.1 | 33.3 |
|  |  | Current | 10.4 | | 10.4 | | 10.2 | 10.4 | 10.4 |
| BMI (median) | | | 26.6 | 26.5 | | 26.5 | | 26.6 | 26.6 |
| Physical activity, frequency/week (median) | | | 11.0 | 11.0 | | 11.0 | | 11.0 | 11.0 |
| Height, cm, men/women (median) | | | 176.0/163.0 | 176.0/163.0 | | 176.0/162.0 | | 176.0/162.0 | 176.0/163.0 |
| Hypertension (%) | | | 52.9 | 52.1 | | 52.6 | | 52.7 | 55.1 |
| Use of lipid-lowering medications (%) | | | 13.1 | 12.2 | | 12.5 | | 13.6 | 13.4 |

**Supplementary Table 6. Total cholesterol concentrations and aortic aneurysm**

|  | Total cholesterol, mmol/L (median) | | | | | | p_trend_ | Continuous |
| --- | --- | --- | --- | --- | --- | --- | --- | --- |
|  | 4.393 | 5.158 | 5.714 | 6.295 | 7.196 |  | | Per 1 mmol/L |
| Participants | 85,983 | 86,004 | 85,822 | 85,916 | 85,885 |  | | 429,610 |
| Cases | 615 | 492 | 448 | 440 | 439 |  | | 2,434 |
| HR (95% CI) | 1.00 | 1.12 (0.99-1.27) | 1.10 (0.97-1.26) | 1.14 (0.99-1.30) | 1.22 (1.07-1.41) | 0.007 | | 1.05 (1.01-1.09) |
| HR (95% CI)^1^ | 1.00 | 1.18 (1.02-1.36) | 1.10 (0.95-1.28) | 1.23 (1.06-1.44) | 1.30 (1.12-1.53) | 0.001 | | 1.06 (1.02-1.11) |
| HR (95% CI), men | 1.00 | 1.10 (0.96-1.26) | 1.14 (0.99-1.32) | 1.17 (1.00-1.36) | 1.37 (1.17-1.61) | <0.001 | | 1.08 (1.03-1.13) |
| HR (95% CI), women | 1.00 | 1.16 (0.86-1.55) | 0.93 (0.68-1.27) | 0.96 (0.70-1.31) | 0.87 (0.64-1.18) | 0.15 | | 0.95 (0.87-1.04) |
| Cases, TAA | 143 | 140 | 126 | 140 | 91 |  | | 640 |
| HR (95% CI) | 1.00 | 1.08 (0.85-1.37) | 0.97 (0.75-1.25) | 1.07 (0.83-1.39) | 0.72 (0.54-0.96) | 0.04 | | 0.91 (0.84-0.99) |
| Cases, AAA | 369 | 250 | 249 | 228 | 273 |  | | 1,369 |
| HR (95% CI) | 1.00 | 1.12 (0.95-1.33) | 1.30 (1.09-1.55) | 1.31 (1.09-1.58) | 1.77 (1.48-2.13) | <0.001 | | 1.17 (1.11-1.23) |
| Cases, UAA | 99 | 96 | 69 | 68 | 73 |  | | 405 |
| HR (95% CI) | 1.00 | 1.19 (0.89-1.59) | 0.89 (0.64-1.23) | 0.90 (0.64-1.26) | 1.02 (0.73-1.43) | 0.64 | | 0.97 (0.88-1.07) |
| Cases, ruptured AA | 37 | 39 | 27 | 21 | 23 |  | | 147 |
| HR (95% CI) | 1.00 | 1.58 (0.98-2.53) | 1.19 (0.69-2.04) | 0.98 (0.54-1.77) | 1.13 (0.62-2.04) | 0.84 | | 1.00 (0.85-1.19) |
| Cases, nonruptured AA | 578 | 453 | 421 | 419 | 416 |  | | 2,287 |
| HR (95% CI) | 1.00 | 1.10 (0.96-1.25) | 1.10 (0.96-1.27) | 1.16 (1.01-1.33) | 1.24 (1.08-1.43) | 0.003 | | 1.05 (1.01-1.10) |
| Cases, AA death | 39 | 33 | 22 | 14 | 14 |  | | 122 |
| HR (95% CI) | 1.00 | 1.29 (0.79-2.10) | 0.93 (0.53-1.65) | 0.63 (0.32-1.23) | 0.68 (0.34-1.34) | 0.09 | | 0.93 (0.78-1.13) |

AA, aortic aneurysm; AAA, abdominal aortic aneurysm; CI, confidence interval; HR, hazard ratio; TAA, thoracic aortic aneurysm; UAA, unspecified aortic aneurysm

Adjusted for age, sex, ethnicity, Townsend deprivation index, education, smoking status and cigarettes/day, BMI categories, frequency of physical activity, height, hypertension, lipid-lowering medication

^1^ Analyses excluding first 5 years of follow-up

**Supplementary Table 7. LDL cholesterol concentrations and aortic aneurysm**

|  | LDL cholesterol, mmol/L (median) | | | | | | p_trend_ | Continuous |
| --- | --- | --- | --- | --- | --- | --- | --- | --- |
|  | 2.558 | 3.135 | 3.567 | 4.020 | 4.713 |  | | Per 1 mmol/L |
| Participants | 86,000 | 85,927 | 85,979 | 85,907 | 85,797 |  | | 429,610 |
| Cases | 551 | 457 | 463 | 475 | 488 |  | | 2,434 |
| HR (95% CI) | 1.00 | 1.14 (1.00-1.30) | 1.25 (1.09-1.43) | 1.30 (1.13-1.49) | 1.39 (1.21-1.60) | <0.001 | | 1.14 (1.08-1.20) |
| HR (95% CI)^1^ | 1.00 | 1.14 (0.99-1.33) | 1.23 (1.06-1.44) | 1.33 (1.14-1.56) | 1.46 (1.25-1.71) | <0.001 | | 1.15 (1.09-1.23) |
| HR (95% CI), men | 1.00 | 1.16 (1.00-1.34) | 1.24 (1.07-1.45) | 1.35 (1.16-1.58) | 1.52 (1.30-1.78) | <0.001 | | 1.18 (1.12-1.26) |
| HR (95% CI), women | 1.00 | 1.07 (0.80-1.44) | 1.23 (0.91-1.66) | 1.10 (0.81-1.50) | 1.08 (0.79-1.46) | 0.81 | | 1.03 (0.92-1.14) |
| Cases, TAA | 127 | 135 | 142 | 142 | 94 |  | | 640 |
| HR (95% CI) | 1.00 | 1.14 (0.89-1.47) | 1.17 (0.91-1.52) | 1.14 (0.88-1.49) | 0.77 (0.58-1.04) | 0.07 | | 0.88 (0.80-0.98) |
| Cases, AAA | 327 | 238 | 234 | 260 | 310 |  | | 1,369 |
| HR (95% CI) | 1.00 | 1.19 (1.00-1.42) | 1.37 (1.14-1.65) | 1.61 (1.34-1.94) | 2.07 (1.72-2.49) | <0.001 | | 1.36 (1.27-1.45) |
| Cases, UAA | 93 | 80 | 81 | 70 | 81 |  | | 405 |
| HR (95% CI) | 1.00 | 1.02 (0.75-1.40) | 1.05 (0.76-1.45) | 0.90 (0.64-1.27) | 1.08 (0.77-1.52) | 0.84 | | 1.01 (0.89-1.16) |
| Cases, ruptured AA | 30 | 34 | 36 | 25 | 22 |  | | 147 |
| HR (95% CI) | 1.00 | 1.74 (1.04-2.90) | 2.06 (1.20-3.51) | 1.46 (0.81-2.65) | 1.33 (0.71-2.48) | 0.62 | | 1.10 (0.88-1.36) |
| Cases, nonruptured AA | 521 | 423 | 427 | 450 | 466 |  | | 2,287 |
| HR (95% CI) | 1.00 | 1.11 (0.97-1.27) | 1.21 (1.05-1.39) | 1.29 (1.12-1.49) | 1.40 (1.21-1.61) | <0.001 | | 1.15 (1.09-1.21) |
| Cases, AA death | 33 | 27 | 29 | 17 | 16 |  | | 122 |
| HR (95% CI) | 1.00 | 1.29 (0.76-2.20) | 1.55 (0.89-2.69) | 0.93 (0.49-1.79) | 0.92 (0.47-1.80) | 0.61 | | 1.04 (0.82-1.31) |

AA, aortic aneurysm; AAA, abdominal aortic aneurysm; CI, confidence interval; HR, hazard ratio; TAA, thoracic aortic aneurysm; UAA, unspecified aortic aneurysm

Adjusted for age, sex, ethnicity, Townsend deprivation index, education, smoking status and cigarettes/day, BMI categories, frequency of physical activity, height, hypertension, lipid-lowering medication

^1^ Analyses excluding first 5 years of follow-up

**Supplementary Table 8. Apolipoprotein B concentrations and aortic aneurysm**

|  | Apolipoprotein B, g/L (median) | | | | | | p_trend_ | Continuous |
| --- | --- | --- | --- | --- | --- | --- | --- | --- |
|  | 0.756 | 0.910 | 1.029 | 1.154 | 1.350 |  | | Per 1 g/L |
| Participants | 86,456 | 86,047 | 85,954 | 85,364 | 85,789 |  | | 429,610 |
| Cases | 470 | 449 | 488 | 458 | 569 |  | | 2434 |
| HR (95% CI) | 1.00 | 1.14 (1.00-1.30) | 1.28 (1.12-1.46) | 1.23 (1.07-1.42) | 1.52 (1.33-1.74) | <0.001 | | 1.76 (1.47-2.10) |
| HR (95% CI)^1^ | 1.00 | 1.10 (0.95-1.28) | 1.28 (1.10-1.49) | 1.24 (1.06-1.45) | 1.57 (1.35-1.83) | <0.001 | | 1.82 (1.49-2.22) |
| HR (95% CI), men | 1.00 | 1.13 (0.97-1.31) | 1.32 (1.13-1.53) | 1.30 (1.11-1.51) | 1.65 (1.42-1.93) | <0.001 | | 2.00 (1.63-2.45) |
| HR (95% CI), women | 1.00 | 1.14 (0.86-1.52) | 1.13 (0.84-1.51) | 1.02 (0.75-1.37) | 1.15 (0.85-1.54) | 0.60 | | 1.17 (0.80-1.71) |
| Cases, TAA | 126 | 123 | 157 | 126 | 108 |  | | 640 |
| HR (95% CI) | 1.00 | 0.98 (0.76-1.26) | 1.21 (0.94-1.54) | 0.95 (0.73-1.23) | 0.80 (0.61-1.06) | 0.09 | | 0.65 (0.45-0.94) |
| Cases, AAA | 255 | 243 | 242 | 258 | 371 |  | | 1,369 |
| HR (95% CI) | 1.00 | 1.28 (1.07-1.53) | 1.41 (1.17-1.69) | 1.62 (1.34-1.95) | 2.37 (1.98-2.83) | <0.001 | | 3.43 (2.72-4.32) |
| Cases, UAA | 84 | 80 | 84 | 71 | 86 |  | | 405 |
| HR (95% CI) | 1.00 | 1.03 (0.76-1.41) | 1.07 (0.78-1.47) | 0.91 (0.65-1.27) | 1.09 (0.79-1.52) | 0.79 | | 1.03 (0.66-1.62) |
| Cases, ruptured AA | 22 | 32 | 43 | 23 | 27 |  | | 147 |
| HR (95% CI) | 1.00 | 1.85 (1.07-3.21) | 2.65 (1.54-4.55) | 1.50 (0.80-2.79) | 1.73 (0.93-3.19) | 0.27 | | 1.64 (0.79-3.41) |
| Cases, nonruptured AA | 448 | 417 | 445 | 435 | 542 |  | | 2,287 |
| HR (95% CI) | 1.00 | 1.10 (0.96-1.26) | 1.22 (1.06-1.40) | 1.22 (1.06-1.41) | 1.51 (1.32-1.74) | <0.001 | | 1.77 (1.47-2.13) |
| Cases, AA death | 23 | 30 | 28 | 21 | 20 |  | | 122 |
| HR (95% CI) | 1.00 | 1.75 (1.00-3.03) | 1.76 (0.99-3.15) | 1.41 (0.75-2.66) | 1.34 (0.69-2.58) | 0.63 | | 1.50 (0.67-3.37) |

AA, aortic aneurysm; AAA, abdominal aortic aneurysm; CI, confidence interval; HR, hazard ratio; TAA, thoracic aortic aneurysm; UAA, unspecified aortic aneurysm

Adjusted for age, sex, ethnicity, Townsend deprivation index, education, smoking status and cigarettes/day, BMI categories, frequency of physical activity, height, hypertension, lipid-lowering medication

^1^ Analyses excluding first 5 years of follow-up

**Supplementary Table 9. Non-HDL cholesterol concentrations and aortic aneurysm**

|  | Non-HDL cholesterol, mmol/L (median) | | | | | | p_trend_ | Continuous |
| --- | --- | --- | --- | --- | --- | --- | --- | --- |
|  | 3.002 | 3.702 | 4.237 | 4.804 | 5.678 |  | | Per 1 mmol/L |
| Participants | 78,712 | 78,763 | 78,581 | 78,615 | 78,741 |  | | 393,412 |
| Cases | 454 | 421 | 427 | 439 | 491 |  | | 2,232 |
| HR (95% CI) | 1.00 | 1.18 (1.03-1.35) | 1.28 (1.11-1.47) | 1.31 (1.13-1.51) | 1.50 (1.29-1.73) | <0.001 | | 1.13 (1.08-1.18) |
| HR (95% CI)^1^ | 1.00 | 1.20 (1.03-1.40) | 1.28 (1.09-1.51) | 1.34 (1.13-1.58) | 1.58 (1.34-1.86) | <0.001 | | 1.14 (1.09-1.20) |
| HR (95% CI), men | 1.00 | 1.18 (1.01-1.38) | 1.27 (1.08-1.49) | 1.39 (1.18-1.63) | 1.61 (1.37-1.90) | <0.001 | | 1.16 (1.11-1.22) |
| HR (95% CI), women | 1.00 | 1.14 (0.84-1.55) | 1.27 (0.93-1.73) | 1.06 (0.77-1.46) | 1.18 (0.86-1.62) | 0.61 | | 1.02 (0.94-1.12) |
| Cases, TAA | 115 | 110 | 134 | 121 | 100 |  | | 580 |
| HR (95% CI) | 1.00 | 0.99 (0.76-1.29) | 1.18 (0.91-1.54) | 1.03 (0.78-1.36) | 0.86 (0.64-1.15) | 0.31 | | 0.92 (0.84-1.01) |
| Cases, AAA | 256 | 220 | 222 | 242 | 317 |  | | 1,257 |
| HR (95% CI) | 1.00 | 1.27 (1.05-1.52) | 1.48 (1.22-1.79) | 1.68 (1.38-2.04) | 2.29 (1.90-2.77) | <0.001 | | 1.31 (1.24-1.38) |
| Cases, UAA | 78 | 90 | 65 | 72 | 71 |  | | 376 |
| HR (95% CI) | 1.00 | 1.27 (0.93-1.74) | 0.92 (0.65-1.31) | 1.00 (0.70-1.42) | 1.00 (0.70-1.43) | 0.58 | | 0.97 (0.87-1.09) |
| Cases, ruptured AA | 23 | 92 | 36 | 23 | 24 |  | | 135 |
| HR (95% CI) | 1.00 | 1.76 (1.00-3.08) | 2.39 (1.36-4.20) | 1.55 (0.82-2.92) | 1.63 (0.86-3.09) | 0.32 | | 1.13 (0.95-1.35) |
| Cases, nonruptured AA | 431 | 392 | 391 | 416 | 467 |  | | 2,097 |
| HR (95% CI) | 1.00 | 1.15 (1.00-1.32) | 1.22 (1.06-1.42) | 1.30 (1.12-1.51) | 1.49 (1.28-1.73) | <0.001 | | 1.13 (1.08-1.18) |
| Cases, AA death | 27 | 27 | 28 | 16 | 15 |  | | 113 |
| HR (95% CI) | 1.00 | 1.43 (0.82-2.48) | 1.63 (0.92-2.90) | 0.94 (0.48-1.85) | 0.89 (0.44-1.80) | 0.50 | | 1.04 (0.86-1.27) |

AA, aortic aneurysm; AAA, abdominal aortic aneurysm; CI, confidence interval; HR, hazard ratio; TAA, thoracic aortic aneurysm; UAA, unspecified aortic aneurysm

Adjusted for age, sex, ethnicity, Townsend deprivation index, education, smoking status and cigarettes/day, BMI categories, frequency of physical activity, height, hypertension, lipid-lowering medication

^1^ Analyses excluding first 5 years of follow-up

**Supplementary Table 10. HDL cholesterol concentrations and aortic aneurysm**

|  | HDL cholesterol, mmol/L (median) | | | | | | p_trend_ | Continuous |
| --- | --- | --- | --- | --- | --- | --- | --- | --- |
|  | 1.020 | 1.232 | 1.413 | 1.624 | 1.972 |  | | Per 1 mmol/L |
| Participants | 78,878 | 78,515 | 78,871 | 78,635 | 78,513 |  | | 393,412 |
| Cases | 810 | 550 | 374 | 288 | 210 |  | | 2,232 |
| HR (95% CI) | 1.00 | 0.84 (0.75-0.94) | 0.68 (0.60-0.77) | 0.65 (0.56-0.74) | 0.57 (0.48-0.67) | <0.001 | | 0.55 (0.48-0.64) |
| HR (95% CI)^1^ | 1.00 | 0.89 (0.78-1.00) | 0.73 (0.64-0.85) | 0.68 (0.58-0.80) | 0.58 (0.48-0.70) | <0.001 | | 0.60 (0.51-0.70) |
| HR (95% CI), men | 1.00 | 0.85 (0.75-0.96) | 0.70 (0.60-0.80) | 0.72 (0.61-0.84) | 0.53 (0.42-0.67) | <0.001 | | 0.55 (0.46-0.65) |
| HR (95% CI), women | 1.00 | 0.82 (0.60-1.11) | 0.64 (0.47-0.87) | 0.51 (0.37-0.70) | 0.53 (0.39-0.73) | <0.001 | | 0.56 (0.42-0.73) |
| Cases, TAA | 143 | 137 | 114 | 93 | 93 |  | | 580 |
| HR (95% CI) | 1.00 | 1.10 (0.87-1.39) | 1.03 (0.80-1.33) | 0.97 (0.74-1.29) | 1.12 (0.83-1.50) | 0.71 | | 1.04 (0.80-1.35) |
| Cases, AAA | 546 | 331 | 176 | 133 | 71 |  | | 1,257 |
| HR (95% CI) | 1.00 | 0.79 (0.69-0.91) | 0.52 (0.44-0.62) | 0.51 (0.42-0.62) | 0.34 (0.26-0.45) | <0.001 | | 0.33 (0.27-0.41) |
| Cases, UAA | 115 | 76 | 83 | 59 | 43 |  | | 376 |
| HR (95% CI) | 1.00 | 0.80 (0.59-1.07) | 1.01 (0.76-1.36) | 0.86 (0.62-1.21) | 0.76 (0.51-1.12) | 0.26 | | 0.90 (0.64-1.25) |
| Cases, ruptured AA | 51 | 42 | 14 | 15 | 13 |  | | 135 |
| HR (95% CI) | 1.00 | 1.00 (0.66-1.52) | 0.39 (0.21-0.71) | 0.49 (0.27-0.91) | 0.49 (0.25-0.96) | 0.003 | | 0.41 (0.22-0.75) |
| Cases, nonruptured AA | 759 | 508 | 360 | 273 | 197 |  | | 2,097 |
| HR (95% CI) | 1.00 | 0.83 (0.74-0.93) | 0.70 (0.62-0.80) | 0.66 (0.57-0.76) | 0.57 (0.48-0.68) | <0.001 | | 0.56 (0.49-0.65) |
| Cases, AA death | 45 | 35 | 12 | 14 | 7 |  | | 113 |
| HR (95% CI) | 1.00 | 1.02 (0.65-1.60) | 0.42 (0.22-0.80) | 0.60 (0.32-1.14) | 0.36 (0.15-0.85) | 0.003 | | 0.37 (0.19-0.73) |

AA, aortic aneurysm; AAA, abdominal aortic aneurysm; CI, confidence interval; HR, hazard ratio; TAA, thoracic aortic aneurysm; UAA, unspecified aortic aneurysm

Adjusted for age, sex, ethnicity, Townsend deprivation index, education, smoking status and cigarettes/day, BMI categories, frequency of physical activity, height, hypertension, lipid-lowering medication

^1^ Analyses excluding first 5 years of follow-up

**Supplementary Table 11. Apolipoprotein A1 concentrations and aortic aneurysm**

|  | Apolipoprotein A, g/L (median) | | | | | | p_trend_ | Continuous |
| --- | --- | --- | --- | --- | --- | --- | --- | --- |
|  | 1.226 | 1.391 | 1.520 | 1.663 | 1.910 |  | | Per 1 g/L |
| Participants | 78,581 | 78,087 | 78,284 | 78,262 | 77,879 |  | | 391,093 |
| Cases | 751 | 561 | 358 | 324 | 228 |  | | 2,222 |
| HR (95% CI) | 1.00 | 0.86 (0.77-0.96) | 0.62 (0.55-0.71) | 0.65 (0.57-0.75) | 0.54 (0.46-0.63) | <0.001 | | 0.39 (0.32-0.47) |
| HR (95% CI)^1^ | 1.00 | 0.89 (0.78-1.00) | 0.67 (0.58-0.77) | 0.68 (0.58-0.79) | 0.54 (0.45-0.65) | <0.001 | | 0.43 (0.35-0.54) |
| HR (95% CI), men | 1.00 | 0.88 (0.78-0.99) | 0.65 (0.57-0.75) | 0.66 (0.56-0.78) | 0.55 (0.44-0.67) | <0.001 | | 0.37 (0.29-0.48) |
| HR (95% CI), women | 1.00 | 0.75 (0.55-1.02) | 0.53 (0.39-0.73) | 0.59 (0.44-0.80) | 0.47 (0.35-0.64) | <0.001 | | 0.38 (0.25-0.57) |
| Cases, TAA | 137 | 139 | 102 | 112 | 88 |  | | 578 |
| HR (95% CI) | 1.00 | 1.09 (0.86-1.38) | 0.87 (0.67-1.13) | 1.06 (0.81-1.38) | 0.93 (0.69-1.25) | 0.60 | | 0.97 (0.68-1.38) |
| Cases, AAA | 500 | 327 | 189 | 143 | 93 |  | | 1,252 |
| HR (95% CI) | 1.00 | 0.79 (0.69-0.91) | 0.54 (0.45-0.64) | 0.48 (0.40-0.59) | 0.38 (0.30-0.49) | <0.001 | | 0.22 (0.17-0.29) |
| Cases, UAA | 109 | 90 | 64 | 65 | 45 |  | | 373 |
| HR (95% CI) | 1.00 | 0.92 (0.70-1.22) | 0.74 (0.54-1.01) | 0.85 (0.61-1.17) | 0.68 (0.47-1.00) | 0.04 | | 0.61 (0.38-0.96) |
| Cases, ruptured AA | 48 | 34 | 23 | 15 | 15 |  | | 135 |
| HR (95% CI) | 1.00 | 0.80 (0.52-1.25) | 0.60 (0.36-1.00) | 0.44 (0.24-0.80) | 0.48 (0.26-0.91) | 0.003 | | 0.30 (0.14-0.66) |
| Cases, nonruptured AA | 703 | 527 | 335 | 309 | 213 |  | | 2,087 |
| HR (95% CI) | 1.00 | 0.86 (0.77-0.97) | 0.63 (0.55-0.72) | 0.67 (0.58-0.77) | 0.54 (0.46-0.64) | <0.001 | | 0.40 (0.33-0.49) |
| Cases, AA death | 43 | 30 | 19 | 11 | 10 |  | | 113 |
| HR (95% CI) | 1.00 | 0.84 (0.53-1.35) | 0.61 (0.35-1.05) | 0.41 (0.20-0.81) | 0.42 (0.20-0.88) | 0.002 | | 0.26 (0.11-0.62) |

AA, aortic aneurysm; AAA, abdominal aortic aneurysm; CI, confidence interval; HR, hazard ratio; TAA, thoracic aortic aneurysm; UAA, unspecified aortic aneurysm

Adjusted for age, sex, ethnicity, Townsend deprivation index, education, smoking status and cigarettes/day, BMI categories, frequency of physical activity, height, hypertension, lipid-lowering medication

^1^ Analyses excluding first 5 years of follow-up

**Supplementary Table 12. Triglyceride concentrations and aortic aneurysm**

|  | Triglycerides, mmol/L (median) | | | | | | p_trend_ | Continuous |
| --- | --- | --- | --- | --- | --- | --- | --- | --- |
|  | 0.787 | 1.118 | 1.470 | 1.956 | 2.981 |  | | Per 1 mmol/L |
| Participants | 86,174 | 85,821 | 85,829 | 85,889 | 85,897 |  | | 429,610 |
| Cases | 275 | 428 | 474 | 558 | 699 |  | | 2,434 |
| HR (95% CI) | 1.00 | 1.18 (1.01-1.37) | 1.09 (0.94-1.27) | 1.11 (0.96-1.29) | 1.23 (1.06-1.42) | 0.022 | | 1.05 (1.01-1.09) |
| HR (95% CI)^1^ | 1.00 | 1.17 (0.99-1.39) | 1.14 (0.97-1.35) | 1.14 (0.96-1.35) | 1.24 (1.05-1.46) | 0.044 | | 1.04 (1.00-1.09) |
| HR (95% CI), men | 1.00 | 1.22 (1.01-1.46) | 1.16 (0.97-1.39) | 1.20 (1.00-1.42) | 1.27 (1.07-1.50) | 0.03 | | 1.05 (1.01-1.09) |
| HR (95% CI), women | 1.00 | 1.11 (0.84-1.46) | 0.94 (0.71-1.26) | 0.90 (0.67-1.22) | 1.15 (0.86-1.55) | 0.49 | | 1.05 (0.95-1.15) |
| Cases, TAA | 99 | 120 | 152 | 132 | 137 |  | | 640 |
| HR (95% CI) | 1.00 | 1.00 (0.76-1.30) | 1.12 (0.86-1.45) | 0.88 (0.67-1.15) | 0.84 (0.64-1.11) | 0.06 | | 0.93 (0.86-1.02) |
| Cases, AAA | 118 | 213 | 238 | 323 | 477 |  | | 1,369 |
| HR (95% CI) | 1.00 | 1.32 (1.05-1.65) | 1.19 (0.95-1.49) | 1.37 (1.10-1.69) | 1.73 (1.40-2.13) | <0.001 | | 1.14 (1.09-1.19) |
| Cases, UAA | 54 | 92 | 84 | 97 | 78 |  | | 405 |
| HR (95% CI) | 1.00 | 1.35 (0.96-1.89) | 1.06 (0.75-1.50) | 1.07 (0.76-1.51) | 0.77 (0.53-1.10) | 0.006 | | 0.86 (0.77-0.96) |
| Cases, ruptured AA | 16 | 32 | 28 | 30 | 41 |  | | 147 |
| HR (95% CI) | 1.00 | 1.42 (0.77-2.59) | 1.01 (0.55-1.89) | 0.93 (0.50-1.73) | 1.15 (0.64-2.10) | 0.93 | | 1.05 (0.90-1.22) |
| Cases, nonruptured AA | 259 | 396 | 446 | 528 | 658 |  | | 2,287 |
| HR (95% CI) | 1.00 | 1.17 (1.00-1.36) | 1.10 (0.94-1.28) | 1.13 (0.97-1.31) | 1.23 (1.06-1.43) | 0.02 | | 1.05 (1.01-1.09) |
| Cases, AA death | 13 | 26 | 20 | 31 | 32 |  | | 122 |
| HR (95% CI) | 1.00 | 1.43 (0.73-2.79) | 0.90 (0.45-1.83) | 1.19 (0.61-2.30) | 1.07 (0.55-2.09) | 0.81 | | 0.97 (0.81-1.16) |

AA, aortic aneurysm; AAA, abdominal aortic aneurysm; CI, confidence interval; HR, hazard ratio; TAA, thoracic aortic aneurysm; UAA, unspecified aortic aneurysm

Adjusted for age, sex, ethnicity, Townsend deprivation index, education, smoking status and cigarettes/day, BMI categories, frequency of physical activity, height, hypertension, lipid-lowering medication

^1^ Analyses excluding first 5 years of follow-up

**Supplementary Table 13. Lipoprotein(a) concentrations and aortic aneurysm (mg/dl)**

|  | Lipoprotein(a), mg/dl (median) | | | | | | p_trend_ | Continuous |
| --- | --- | --- | --- | --- | --- | --- | --- | --- |
|  | 4.372 | 6.830 | 11.344 | 24.133 | 61.573 |  | | Per 50 mg/dl |
| Participants | 69,028 | 68,942 | 69,055 | 68,941 | 68,992 |  | | 344,958 |
| Cases | 362 | 366 | 357 | 395 | 450 |  | | 1,930 |
| HR (95% CI) | 1.00 | 1.04 (0.90-1.20) | 1.02 (0.88-1.19) | 1.19 (1.03-1.37) | 1.34 (1.17-1.54) | <0.001 | | 1.27 (1.16-1.39) |
| HR (95% CI)^1^ | 1.00 | 1.00 (0.85-1.18) | 1.00 (0.85-1.18) | 1.16 (0.99-1.37) | 1.32 (1.12-1.54) | <0.001 | | 1.27 (1.15-1.41) |
| HR (95% CI), men | 1.00 | 1.04 (0.88-1.22) | 1.09 (0.93-1.29) | 1.25 (1.06-1.47) | 1.40 (1.19-1.63) | <0.001 | | 1.31 (1.18-1.46) |
| HR (95% CI), women | 1.00 | 1.05 (0.77-1.44) | 0.82 (0.59-1.13) | 1.03 (0.76-1.40) | 1.14 (0.84-1.55) | 0.11 | | 1.12 (0.92-1.37) |
| Cases, TAA | 103 | 109 | 94 | 96 | 109 |  | | 511 |
| HR (95% CI) | 1.00 | 1.09 (0.83-1.44) | 0.96 (0.72-1.28) | 1.03 (0.77-1.36) | 1.15 (0.87-1.51) | 0.31 | | 1.04 (0.86-1.26) |
| Cases, AAA | 181 | 202 | 216 | 227 | 256 |  | | 1,082 |
| HR (95% CI) | 1.00 | 1.14 (0.93-1.40) | 1.25 (1.02-1.52) | 1.34 (1.09-1.63) | 1.53 (1.26-1.85) | <0.001 | | 1.36 (1.20-1.54) |
| Cases, UAA | 76 | 53 | 47 | 65 | 80 |  | | 321 |
| HR (95% CI) | 1.00 | 0.72 (0.51-1.03) | 0.66 (0.46-0.95) | 0.96 (0.69-1.34) | 1.12 (0.82-1.55) | 0.02 | | 1.31 (1.05-1.65) |
| Cases, ruptured AA | 20 | 21 | 23 | 28 | 24 |  | | 116 |
| HR (95% CI) | 1.00 | 1.20 (0.54-2.68) | 1.44 (0.67-3.10) | 1.33 (0.60-2.93) | 1.06 (0.46-2.45) | 0.80 | | 1.23 (0.84-1.81) |
| Cases, nonruptured AA | 342 | 345 | 334 | 367 | 426 |  | | 1,814 |
| HR (95% CI) | 1.00 | 1.04 (0.89-1.20) | 1.02 (0.88-1.19) | 1.18 (1.01-1.36) | 1.35 (1.17-1.55) | <0.001 | | 1.27 (1.15-1.40) |
| Cases, AA death | 15 | 13 | 13 | 22 | 31 |  | | 94 |
| HR (95% CI) | 1.00 | 0.90 (0.43-1.90) | 0.90 (0.43-1.89) | 1.58 (0.82-3.06) | 2.27 (1.22-4.20) | <0.001 | | 2.10 (1.45-3.05) |

AA, aortic aneurysm; AAA, abdominal aortic aneurysm; CI, confidence interval; HR, hazard ratio; TAA, thoracic aortic aneurysm; UAA, unspecified aortic aneurysm

Adjusted for age, sex, ethnicity, Townsend deprivation index, education, smoking status and cigarettes/day, BMI categories, frequency of physical activity, height, hypertension, lipid-lowering medication

^1^ Analyses excluding first 5 years of follow-up

**Supplementary Table 14. List of excluded studies and exclusion reasons**

| Exclusion reason | Reference number |
| --- | --- |
| Abstract | (1-8) |
| Case-control study | (9-20) |
| Comment | (21) |
| Cross-sectional study | (22-33) |
| Duplicate | (34-37) |
| Ecological study | (38-41) |
| Editorial | (42;43) |
| GWAS | (44-46) |
| Letter | (47;48) |
| Meta-analysis | (49-54) |
| Modeling study | (55) |
| Mendelian randomization study | (56-73) |
| No risk estimates | (74) |
| Not relevant exposure | (75-81) |
| Not relevant outcome | (82;83) |
| Patients | (84-91) |
| Preprint | (92) |
| Review | (93-98) |

Reference List

1. Tang W, Alonso A, Lutsey PL et al. Associations between middle-age risk factors and risk of asymptomatic abdominal aortic aneurysm: The atherosclerosis risk in communities (ARIC) study. Circulation Conference: American Heart Association's Epidemiology and Prevention/Nutrition, Physical Activity, and Metabolism 2014;25.

2. Weng L-C, Lutsey PL, Alonso A et al. Association between plasma low-density lipoprotein cholesterol level and risk of abdominal aortic aneurysm in ARIC: A mendelian randomization study. Circulation Conference: American Heart Association's Epidemiology and Prevention/Nutrition, Physical Activity, and Metabolism 2014;25.

3. Moayyeri A, Patel R, Pujades RM et al. Low levels of LDL-cholesterol and incidence of a wide range of cardiovascular diseases: A linked electronic health records cohort of 550,000 people. European Heart Journal Conference: European Society of Cardiology, ESC Congress 2016;01.

4. Safarova MS, Fan X, Jarvik GP et al. A phenome-wide association study to assess pleiotropic effects of LPA. Circulation Conference: Resuscitation Science Symposium, ReSS 2017;November.

5. Fattahi N, Rosenblad A, Kragsterman B, Hultgren R. Risk Factors in 50-year-old Men Predicting Development of Abdominal Aortic Aneurysm - A prospective Cohort Study with 15 Years of Follow-up. European Journal of Vascular and Endovascular Surgery Conference: The European Society for Vascular Surgery 33rd Annual Meeting 2019;December.

6. Van Den BM, Snaterse M, Van Trier TJ et al. Heterogeneous associations of traditional atherosclerotic risk factors and long-term events in different arterial territories: the EPIC Norfolk prospective population cohort. European Heart Journal Conference: European Society of Cardiology Congress, ESC 2024;01.

7. Rikhi RR, McParland J, Bancks M et al. Association between lp(A) And aortic aneurysm: The uk biobank. Journal of the American College of Cardiology Conference: American College of Cardiology, (ACC) Meeting 2025;01.

8. Van Den BM, Snaterse M, Martens FMAC, Boekholdt SM, Jorstad HT. Clustering of atherosclerotic risk factors associated with differential complications across the arterial territories in the EPIC-Norfolk Cohort. European Journal of Preventive Cardiology Conference: ESC Preventive Cardiology Congress 2025;01.

9. Norrgard O, Angquist K-A, Johnson O. Familial aortic aneurysms: Serum concentrations of triglyceride, cholesterol, HDL-cholesterol and (VLDL + LDL)-cholesterol. British Journal of Surgery 72(2) (pp 113-116), 1985;1985.

10. Norrgard O, Angquist KA, Dahlen G. High concentrations of Lp(a) lipoprotein in serum are common among patients with abdominal aortic aneurysms. Int Angiol 1988;7:46-9.

11. Franks PJ, Edwards RJ, Greenhalgh RM, Powell JT. Risk factors for abdominal aortic aneurysms in smokers. Eur J Vasc Endovasc Surg 1996;11:487-92.

12. Cole CW, Hill GB, Millar WJ, Laupacis A, Johnston KW. Selective screening for abdominal aortic aneurysm. Chronic diseases in Canada 17(2) (pp 51-55), 1996;1996.

13. Blanchard JF, Armenian HK, Friesen PP. Risk factors for abdominal aortic aneurysm: results of a case-control study. Am J Epidemiol 2000;151:575-83.

14. Schillinger M, Domanovits H, Ignatescu M et al. Lipoprotein (a) in patients with aortic aneurysmal disease. J Vasc Surg 2002;36:25-30.

15. Jones GT, van Rij AM, Cole J et al. Plasma lipoprotein(a) indicates risk for 4 distinct forms of vascular disease. Clin Chem 2007;53:679-85.

16. Chen XF, Tang LJ, Jiang JJ et al. Increased levels of lipoprotein(a) in non-smoking aortic dissection patients. Clin Exp Med 2008;8:123-7.

17. Rizzo M, Krayenbuhl PA, Pernice V, Frasheri A, Battista RG, Berneis K. LDL size and subclasses in patients with abdominal aortic aneurysm. Int J Cardiol 2009;134:406-8.

18. Ahnstrom J, Gottsater A, Lindblad B, Dahlback B. Plasma concentrations of apolipoproteins A-I, B and M in patients with abdominal aortic aneurysms. Clin Biochem 2010;43:407-10.

19. Papagrigorakis E, Iliopoulos D, Asimacopoulos PJ et al. Lipoprotein(a) in plasma, arterial wall, and thrombus from patients with aortic aneurysm. Clin Genet 1997;52:262-71.

20. Moxon JV, Behl-Gilhotra R, Morton SK et al. Plasma Low-density Lipoprotein Receptor-related Protein 1 Concentration is not Associated with Human Abdominal Aortic Aneurysm Presence. Eur J Vasc Endovasc Surg 2015;50:466-73.

21. Mukamal KJ. Risk factors for aortic aneurysm. Epidemiology 2001;12:752.

22. McConathy WJ, Alaupovic P, Woolcock N, Laing SP, Powell J, Greenhalgh R. Lipids and apolipoprotein profiles in men with aneurysmal and stenosing aorto-iliac atherosclerosis. European Journal of Vascular Surgery 3(6) (pp 511-514), 1989;1989.

23. Louwrens HD, Adamson J, Powell JT, Greenhalgh RM. Risk factors for atherosclerosis in men with stenosing or aneurysmal disease of the abdominal aorta. International Angiology 12(1) (pp 21-24), 1993;1993.

24. Pleumeekers HJ, Hoes AW, van der DE et al. Aneurysms of the abdominal aorta in older adults. The Rotterdam Study. Am J Epidemiol 1995;142:1291-9.

25. Simoni G, Gianotti A, Ardia A, Baiardi A, Galleano R, Civalleri D. Screening study of abdominal aortic aneurysm in a general population: lipid parameters. Cardiovasc Surg 1996;4:445-8.

26. Mattes E, Davis TM, Yang D, Ridley D, Lund H, Norman PE. Prevalence of abdominal aortic aneurysms in men with diabetes. Med J Aust 1997;166:630-3.

27. Singh K, Bonaa KH, Jacobsen BK, Bjork L, Solberg S. Prevalence of and risk factors for abdominal aortic aneurysms in a population-based study : The Tromso¸ Study. Am J Epidemiol 2001;154:236-44.

28. Hobbs SD, Claridge MW, Quick CR, Day NE, Bradbury AW, Wilmink AB. LDL cholesterol is associated with small abdominal aortic aneurysms. Eur J Vasc Endovasc Surg 2003;26:618-22.

29. Golledge J, van BF, Jamrozik K, McCann M, Norman PE. Association between serum lipoproteins and abdominal aortic aneurysm. Am J Cardiol 2010;105:1480-4.

30. Chun KC, Teng KY, Chavez LA et al. Risk factors associated with the diagnosis of abdominal aortic aneurysm in patients screened at a regional Veterans Affairs health care system. Ann Vasc Surg 2014;28:87-92.

31. Carter JL, Morris DR, Sherliker P et al. Sex-Specific Associations of Vascular Risk Factors With Abdominal Aortic Aneurysm: Findings From 1.5 Million Women and 0.8 Million Men in the United States and United Kingdom. J Am Heart Assoc 2020;9:e014748.

32. Lin W, Luo S, Li W et al. Association between the non-HDL-cholesterol to HDL- cholesterol ratio and abdominal aortic aneurysm from a Chinese screening program. Lipids Health Dis 2023;22:187.

33. Preechasuk L, Kongmalai T, Lapinee V, Pratumvinit B, Thongtang N. The Association of Lipoprotein(a) Levels with Atherosclerotic Cardiovascular Disease in Thailand: A Cross-Sectional Study. Vascular Health and Risk Management 21(pp 813-822), 2025;2025.

34. Oyenuga AO, Folsom AR, Lutsey PL, Tang W. Association of Life's Simple 7 with reduced clinically manifest abdominal aortic aneurysm: The ARIC study. Vascular Medicine (United Kingdom) 24(3) (pp 224-229), 2019;01.

35. Nyronning LÃ, Skoog P, Videm V, Mattsson E. Is the aortic size index relevant as a predictor of abdominal aortic aneurysm? A population-based prospective study: the Tromsø study. Scand Cardiovasc J 2020;54:130-7.

36. Peng Z, Qiu P, Guo H et al. Association between high-density lipoprotein cholesterol and risk of abdominal aortic aneurysm among males and females aged 60Â years and over. J Vasc Surg 2025;81:894-904.

37. van den Bogaart M, Snaterse M, Van Trier TJ et al. Differences in long-term impact of traditional risk factors for atherosclerosis on different arterial territories Insights from over 20 years of follow up in the EPIC-Norfolk prospective population study. Eur J Prev Cardiol 2025.

38. Sidloff DA, Stather PW, Dattani N et al. The association of trends in classic cardiovascular risk factors with global trends in abdominal aortic aneurysm mortality. Circulation Conference: American Heart Association 2013;26.

39. Sidloff D, Choke E, Stather P, Bown M, Thompson J, Sayers R. Mortality from thoracic aortic diseases and associations with cardiovascular risk factors. Circulation 2014;130:2287-94.

40. Sidloff D, Stather P, Dattani N et al. Aneurysm global epidemiology study public health measures can further reduce abdominal aortic aneurysm mortality. Circulation 129(7) (pp 747-753), 2014;18.

41. Persson SE, Boman K, Wanhainen A, Carlberg B, Arnerlov C. Decreasing prevalence of abdominal aortic aneurysm and changes in cardiovascular risk factors. J Vasc Surg 2017;65:651-8.

42. Alexandrou AT, Tsimikas S. Elevated Lp(a) and Abdominal Aortic Aneurysm. Angiology 2017;68:96-8.

43. Beckman JA, Sullivan AE. Lipoprotein(a), Peripheral Artery Disease, and Abdominal Aortic Aneurysm: The Next Frontier or Another Risk Enhancer? J Am Coll Cardiol 2023;82:2277-9.

44. Roychowdhury T, Klarin D, Levin MG et al. Genome-wide association meta-analysis identifies risk loci for abdominal aortic aneurysm and highlights PCSK9 as a therapeutic target. Nat Genet 2023;55:1831-42.

45. Zheng S, Tsao PS, Pan C. Shared Genetic Susceptibility between Abdominal Aortic Aneurysm and Cardiometabolic Traits. medRxiv (no pagination), 2023;08.

46. Zheng S, Tsao PS, Pan C. Abdominal aortic aneurysm and cardiometabolic traits share strong genetic susceptibility to lipid metabolism and inflammation. Nat Commun 2024;15:5652.

47. Craig WY, Kloza EM, Haddow JE, Hitchcock JL, Eldrup-Jorgensen J. Lipoprotein(a) levels in peripheral atherosclerotic disease. J Vasc Surg 1995;21:541-2.

48. Li D. Challenges and future directions in high-density lipoprotein cholesterol as a biomarker for abdominal aortic aneurysm risk. J Vasc Surg 2025;82:704.

49. Takagi H, Manabe H, Kawai N, Goto SN, Umemoto T. Circulating lipoprotein(a) concentrations and abdominal aortic aneurysm presence. Interact Cardiovasc Thorac Surg 2009;9:467-70.

50. Takagi H, Manabe H, Umemoto T. A meta-analysis of association between serum lipoproteins and abdominal aortic aneurysm. Am J Cardiol 2010;106:753-4.

51. Takagi H, Manabe H, Kawai N, Goto SN, Umemoto T. Serum high-density and low-density lipoprotein cholesterol is associated with abdominal aortic aneurysm presence: a systematic review and meta-analysis. Int Angiol 2010;29:371-5.

52. Stather PW, Sidloff DA, Dattani N et al. Meta-analysis and meta-regression analysis of biomarkers for abdominal aortic aneurysm. Br J Surg 2014;101:1358-72.

53. Kotani K, Sahebkar A, Serban MC et al. Lipoprotein(a) Levels in Patients With Abdominal Aortic Aneurysm. Angiology 2017;68:99-108.

54. Lampsas S, Oikonomou E, Pantelidis P et al. Lipoprotein (a) Levels and Abdominal Aortic Aneurysm. A Systematic Review and Meta-analysis. Curr Pharm Des 2022;28:3492-9.

55. Wang Z, You Y, Yin Z et al. Burden of Aortic Aneurysm and Its Attributable Risk Factors from 1990 to 2019: An Analysis of the Global Burden of Disease Study 2019. Front Cardiovasc Med 2022;9:901225.

56. Weng LC, Roetker NS, Lutsey PL et al. Evaluation of the relationship between plasma lipids and abdominal aortic aneurysm: A Mendelian randomization study. PLoS One 2018;13:e0195719.

57. Allara E, Morani G, Carter P et al. Genetic Determinants of Lipids and Cardiovascular Disease Outcomes: A Wide-Angled Mendelian Randomization Investigation. Circ Genom Precis Med 2019;12:e002711.

58. Larsson SC, Gill D, Mason AM et al. Lipoprotein(a) in Alzheimer, Atherosclerotic, Cerebrovascular, Thrombotic, and Valvular Disease: Mendelian Randomization Investigation. Circulation 141(22) (pp 1826-1828), 2020;02.

59. Chen Y, Huang M, Xuan Y et al. Association between Lipid Levels and Risk for Different Types of Aneurysms: A Mendelian Randomization Study. J Pers Med 2021;11.

60. Satterfield BA, Dikilitas O, Safarova MS et al. Associations of Genetically Predicted Lp(a) (Lipoprotein [a]) Levels With Cardiovascular Traits in Individuals of European and African Ancestry. Circ Genom Precis Med 2021;14:e003354.

61. Gao Q, Tan JS, Fan L, Wang X, Hua L, Cai J. Causal associations between disorders of lipoprotein metabolism and ten cardiovascular diseases. Front Cell Dev Biol 2022;10:1023006.

62. Wang S, Zha L, Chen J et al. The relationship between lipoprotein(a) and risk of cardiovascular disease: a Mendelian randomization analysis. Eur J Med Res 2022;27:211.

63. Cupido AJ, Asselbergs FW, Schmidt AF, Hovingh GK. Low-Density Lipoprotein Cholesterol Attributable Cardiovascular Disease Risk Is Sex Specific. J Am Heart Assoc 2022;11:e024248.

64. Georgakis MK, Malik R, Burgess S, Dichgans M. Additive Effects of Genetic Interleukin-6 Signaling Downregulation and Low-Density Lipoprotein Cholesterol Lowering on Cardiovascular Disease: A 2Ã—2 Factorial Mendelian Randomization Analysis. J Am Heart Assoc 2022;11:e023277.

65. Li R, Zhang C, Du X, Chen S. Genetic Association between the Levels of Plasma Lipids and the Risk of Aortic Aneurysm and Aortic Dissection: A Two-Sample Mendelian Randomization Study. J Clin Med 2023;12.

66. Pham K, Mulugeta A, Lumsden A, Hyppnen E. Genetically instrumented LDL-cholesterol lowering and multiple disease outcomes: A Mendelian randomization phenome-wide association study in the UK Biobank. British Journal of Clinical Pharmacology 2023;89:2992-3004.

67. Wu Z, Luo S, Cai D et al. The causal relationship between metabolic syndrome and its components and cardiovascular disease: A mendelian randomization study. Diabetes Res Clin Pract 2024;211:111679.

68. Liu C, Peng J, Liu Y, Peng Y, Ma Q. Genetic evidence for lifestyle and cardiometabolic factors on the risk of aortic aneurysms: A comprehensive Mendelian randomization study. Atherosclerosis 2024;397:118572.

69. Zhang T, Li Y, Liu X et al. Relationship of Apolipoprotein B to Aortic Aneurysm: Frailty Index as a Partial Mediator - a Mendelian Randomization Study. Kardiologiia 2025;65:57-63.

70. Sharma P, Judy R, Yuan S et al. Elevated Lipoprotein (a) Is Associated With Increased Risk Of Abdominal Aortic Aneurysm. Arteriosclerosis, Thrombosis, and Vascular Biology Conference: American Heart Association's Vascular Discovery: From Genes to Medicine 2024;01.

71. Ibrahim M, Thanigaimani S, Singh TP, Morris D, Golledge J. Systematic review and Meta-Analysis of Mendelian randomisation analyses of Abdominal aortic aneurysms. Int J Cardiol Heart Vasc 2021;35:100836.

72. Harrison SC, Holmes MV, Burgess S et al. Genetic Association of Lipids and Lipid Drug Targets With Abdominal Aortic Aneurysm: A Meta-analysis. JAMA Cardiol 2018;3:26-33.

73. Luo C, Zhao Y, Zhang J, Ma Q. The association of metabolic syndrome with aortic aneurysm: a two-sample Mendelian randomization study. Eur J Med Res 2025;30:815.

74. Alcorn HG, Wolfson SK, Jr., Sutton-Tyrrell K, Kuller LH, O'Leary D. Risk factors for abdominal aortic aneurysms in older adults enrolled in The Cardiovascular Health Study. Arterioscler Thromb Vasc Biol 1996;16:963-70.

75. Lederle FA, Larson JC, Margolis KL et al. Abdominal aortic aneurysm events in the women's health initiative: cohort study. BMJ 2008;337:a1724.

76. Acosta S, Taimour S, Gottsater A et al. Lp-PLA(2) activity and mass for prediction of incident abdominal aortic aneurysms: A prospective longitudinal cohort study. Atherosclerosis 2017;262:14-8.

77. Stackelberg O, Wolk A, Eliasson K et al. Lifestyle and risk of screening-detected abdominal aortic aneurysm in men. Journal of the American Heart Association 6(5) (no pagination), 2017;e004725.

78. Kubota Y, Folsom AR, Pankow JS, Wagenknecht LE, Tang W. Diabetes-related factors and abdominal aortic aneurysm events: the Atherosclerotic Risk in Communities Study. Ann Epidemiol 2018;28:102-6.

79. Welsh P, Welsh CE, Jhund PS et al. Derivation and Validation of a 10-Year Risk Score for Symptomatic Abdominal Aortic Aneurysm: Cohort Study of Nearly 500 000 Individuals. Circulation 2021;144:604-14.

80. Acosta S, Fatemi S, Zarrouk M, Gottsater A. Novel Plasma Biomarkers Associated with Future Peripheral Atherosclerotic Disease and Abdominal Aortic Aneurysm-Insights from Contemporary Prospective Studies from the Malmo Diet and Cancer Study. Biomolecules 2024;14.

81. Sode BF, Nordestgaard BG, GrÃ¸nbÃ¦k M, Dahl M. Tobacco smoking and aortic aneurysm: two population-based studies. Int J Cardiol 2013;167:2271-7.

82. Burchfiel CM, Laws A, Benfante R et al. Combined effects of HDL cholesterol, triglyceride, and total cholesterol concentrations on 18-year risk of atherosclerotic disease. Circulation 1995;92:1430-6.

83. Pham MHC, Sigvardsen PE, Fuchs A et al. Aortic aneurysms in a general population cohort: prevalence and risk factors in men and women. European Heart Journal Cardiovascular Imaging 25(9) (pp 1235-1243), 2024;01.

84. Ljungberg J, Johansson B, Engstrom KG et al. Traditional Cardiovascular Risk Factors and Their Relation to Future Surgery for Valvular Heart Disease or Ascending Aortic Disease: A Case-Referent Study. J Am Heart Assoc 2017;6.

85. Golledge J, Rowbotham S, Velu R et al. Association of serum lipoprotein (A) with the requirement for a peripheral artery operation and the incidence of major adverse cardiovascular events in people with peripheral artery disease. Journal of the American Heart Association 9(6) (no pagination), 2020;e015355.

86. Brown LC, Powell JT. Risk factors for aneurysm rupture in patients kept under ultrasound surveillance. UK Small Aneurysm Trial Participants. Ann Surg 1999;230:289-96.

87. Powell JT, Brown LC. The natural history of abdominal aortic aneurysms and their risk of rupture. Acta Chir Belg 2001;101:11-6.

88. Sun T, Zhang H, Cheng Y et al. A two-year follow-up for Chinese patients with abdominal aortic aneurysm undergoing open/endovascular repair. Chin Med J (Engl ) 2014;127:457-61.

89. Rustempasic N, Semi S. Correlation of Atherosclerotic Risk Factors with the Size of Abdominal Aortic Aneurysm (AAA). Mater Sociomed 2019;31:273-6.

90. Liu S, Long C, Hong Y, Gu X, Weng R, Zhong Z. Prevalence of risk factors associated with rupture of abdominal aortic aneurysm (AAA): a single center retrospective study. PeerJ 11(no pagination), 2023;e15752.

91. Cronenwett JL, Murphy TF, Zelenock GB. Actuarial analysis of variables associated with rupture of small abdominal aortic aneurysms. Surgery 98(3) (pp 472-483), 1985;1985.

92. Sharma P, Judy R, Yuan S et al. Lipoprotein (a) is associated with increased risk of Abdominal Aortic Aneurysm. medRxiv 2024.

93. Blanchard JF. Epidemiology of abdominal aortic aneurysms. Epidemiol Rev 1999;21:207-21.

94. Nana P, Dakis K, Brodis A, Spanos K, Kouvelos G. Circulating biomarkers for the prediction of abdominal aortic aneurysm growth. Journal of Clinical Medicine 10(8) (no pagination), 2021;1718.

95. Ibrahim M, Thanigaimani S, Singh TP, Morris D, Golledge J. Systematic Review and Meta-Analysis of Mendelian Randomisation Analyses of Abdominal Aortic Aneurysms. Journal of Vascular Surgery Conference: 2021;November.

96. Lu HS, Temel RE, Levin MG, Damrauer SM, Daugherty A. Research Advances in Abdominal Aortic Aneurysms: Triglyceride-Rich Lipoproteins as a Therapeutic Target. Arterioscler Thromb Vasc Biol 2024;44:1171-4.

97. Thomas PE, Vedel-Krogh S, Kamstrup PR. High lipoprotein(a) is a risk factor for peripheral artery disease, abdominal aortic aneurysms, and major adverse limb events. Curr Opin Cardiol 2024;39:511-9.

98. Ferreira HB, Trindade F, Nogueira-Ferreira R et al. Lipidomic insights on abdominal aortic aneurysm and peripheral arterial disease. J Mol Med (Berl) 2025;103:365-80.

**Supplementary Table 15: Characteristics of studies included in the systematic review of blood lipids and risk of aortic aneurysm.**

| **First author, publication year, location, reference** | **Study name** | **Study period, follow-up duration** | **Number of participants, sex, age, number of cases** | **Outcome(s)** | **Exposure(s) of interest** | **Comparison (contrast or metric of blood lipids)** | **RR (95% CI)** | **Adjustment for covariates** |
| --- | --- | --- | --- | --- | --- | --- | --- | --- |
| Strachan DP, 1991, United Kingdom | The Whitehall Study | 1967-1969 - 1987, 18 years follow-up | 18403 men, age 40-64 years: 99 aortic aneurysm deaths | AA | Plasma cholesterol | Per 1 mmol/l | 0.96 (0.78-1.18) | Age, diastolic blood pressure, smoking status, type of tobacco, inhalation behavior |
| Reed D et al, 1992, USA | Honolulu Heart Program | 1965-1968 - 1988, 20 years follow-up | 7682 men, age 46-66 years: 151 aortic aneurysm cases | AA | Serum cholesterol  Serum triglycerides | Per 94 mg/dl  Per 275 mg/dl | 2.32 (1.62-3.32)*  1.07 (0.85-1.35) | Systolic blood pressure, cigarette pack-years, height |
| Goldberg RJ et al, 1995, USA | Honolulu Heart Program | 1965-1988, 23 years follow-up | 2710 men, age 55-64 years: 119 aortic aneurysm cases | AA | Serum cholesterol  Serum triglycerides | <5 mmol/L  5-5.5  5.6-6.2  >=6.2  <1.37mmol/L  1.37-1.96  1.97-2.91  >=2.92 | 1.00  1.18 (0.46-3.01)  1.18 (0.46-3.02)  2.80 (1.22-6.42)  1.00  0.96 (0.41-2.24)  1.16 (0.51-2.67)  0.81 (0.33-1.97) | Ventricular rate, Body mass index, Systolic blood pressure, serum glucose, serum uric acid, hematocrit, forced expiratory volume, physical activity index, cigarette smoking (no of cigarettes per day), alcohol consumption |
| Lee AJ et al, 1997, United Kingdom | The Edinburgh Artery Study | ~1987-1994, 5 years follow-up | 1592 men and women, age 55-74 years: 40 abdominal aortic aneurysm cases | AAA | Total cholesterol | Per 1 mmol/L | 1.17 (0.90-1.53) | Concurrent atherosclerotic disease, ankle brachial pressure index (ABPI), sex, age |
| Watt HC et al, 1998, United Kingdom | British United Provident Association (BUPA) study | 1975-1982 - 1993, 13 years follow-up | 21520 men, age 35-64 years: 30 abdominal aortic aneurysm deaths  150 controls | AAA | Cholesterol  Cholesterol  Triglycerides  Apolipoprotein B  Apolipoprotein A1  LipoproteinA  Apolipoprotein A1 | 90 vs. 10 percentile  Per 0.6 mmol/L  90 vs. 10 percentile  90 vs. 10 percentile  90 vs. 10 percentile  90 vs. 10 percentile  Per 0.12 mmol/L | 1.50 (0.55-4.30)  1.10 (0.88-1.36)  12 (3.8-37.0)  5.5 (1.8-17.0)  0.15 (0.04-0.56)  1.60 (0.60-3.00)  0.64 (0.47-0.87) | Age, duration of storage of serum, number of serum freeze-thaw cycles, and smoking |
| Törnwall MF et al, 2001, Finland | Alpha-Tocopherol, Beta-Carotene Cancer Prevention Study | 1985-1993, 5.8 years follow-up | 29122 male smokers, age 50-69 years: 181 abdominal aortic aneurysm cases | AAA | Total cholesterol  HDL cholesterol | <5.0 mmol/L  >5.0–6.5  >6.5  <0.9 mmol/L  >0.9-1.5  >1.5 | 1.00  1.30 (0.77-2.20)  1.81 (1.07-3.05)  1.00  0.40 (0.29-0.56)  0.15 (0.07-0.31) | Age, smoking years, cigarettes/day, systolic blood pressure, diastolic blood pressure, BMI, education, exercise, diabetes mellitus, alpha-tocopherol and beta-carotene supplementation group, mutual adjustment between total and HDL cholesterol |
| Rodin MB et al, 2003, USA | Chicago Heart Association Detection Project in Industry cohort | 1967-1973 - 1992, 30 years follow-up | 19274 men and women, age 40-64 years: 418 abdominal aortic aneurysm cases | AAA | Serum cholesterol | Per 1 SD (40mg/dL) | 1.30-1.34 | Age, gender, systolic BP, Diastolic BP, antihypertensive drug treatment, current smoker vs never smoker, ex-smoker vs never smoked, cigarettes/d. height |
| Lindblad B et al, 2005, Sweden | Malmo Preventive Study | 1974 - 1991 - 2002, 21 years follow-up | Nested case-control study: 126 abdominal aortic aneurysm cases  126 controls  Men, mean age 43.7 years | AAA | Serum triglycerides  Serum cholesterol | Per 0.7 mmol/L  Per 1 mmol/L | 1.28 (0.92-1.79)  1.45 (1.05-1.99) | Age |
| Wanhainen A et al, 2005, Sweden | Vasterbotten Intervention Program | 1984-1994, 12 years follow-up | Nested case-control study: 35 abdominal aortic aneurysm cases  140 controls  Men and women, age 60 years | AAA | Total cholesterol  LDL cholesterol  Triglycerides | Per 1 mmol/L  Per 1 mmol/L  Per 1 mmol/L | 1.90 (1.27-2.83)  2.32 (1.23-4.36)  1.91 (1.18-3.09) | Age, sex |
| Iribarren C et al, 2007, USA | Kaiser Permanente Multiphasic Health Checkups | 1965-1970 - 2003, 13 years follow-up | 104813 men and women, age ≥18 years: 605 abdominal aortic aneurysm cases | AAA | Serum total cholesterol | ≤199 mg/dL  200-239  ≥240 | 1.00  4.36 (1.85-10.28)  4.94 (2.09-11.68) | Age, sex, race, education, cigarette smoking status and packs/day, alcohol, height, sagital abdominal diameter |
| Forsdahl SH et al, 2009, Norway | The Tromsø Study 5 | 1994-2001, 7 years follow-up | 4345 men and women, age 25-82 years: 119 abdominal aortic aneurysm cases | AAA | Serum total cholesterol  Serum HDL cholesterol | <5.85mmol/L  5.86-6.69  6.70-7.54  >7.55  <1.25mmol/L  1.25-1.51  1.52-1.82  >1.83 | 1.00  0.97 (0.54-1.75)  1.27 (0.72-2.25)  2.11 (1.23-3.64)  3.25 (1.68–6.27)  2.66 (1.15-4.45)  1.61 (0.79-3.28)  1.0 | Age, sex |
| Landenhed M et al, 2015, Sweden | Malmo Diet and Cancer Study | 1991-1996 - NA, 16 years follow-up | 30412 men and women, age 41-73 years: 127 abdominal aortic aneurysm cases  45 thoracic aortic aneurysm cases | AAA  TAA | ApolipoproteinB/apolipoprotein A1, AAA  Apolipoprotein A1  Apolipoprotein B  ApolipoproteinB/apolipoprotein A1, TAA  Apolipoprotein A1  Apolipoprotein B | ≥0.88  <133 mg/dL  ≥128 mg/dL  ≥0.88  <133 mg/dL  ≥128 mg/dL | 2.48 (1.73-3.54)  1.95 (1.36-2.79)  1.59 (1.08-2.33)  1.22 (0.63-2.39)  1.33 (0.72-2.47)  1.73 (0.92-3.25) | Age, sex, hypertension, apoA1 |
| Tang W et al, 2016, USA | Atherosclerosis Risk in Communities study (ARIC) | 1987-2013, 22.5 years median | 5911 men and women, mean age 54.2 years, 665 abdominal aortic aneurysm cases | AAA | HDL-C  LDL-C  Total Cholesterol  Triglycerides | <42.4 mg/dl  42.4-55.9  56.0-163.0  <119.6 mg/dl  119.6-151.8  151.9-504.6  <194.7 mg/dl  195.7-228.6  229.6-593.1  <88 mg/dl  89-136  137-1926 | 1.00  0.67 (0.55-0.81)  0.48 (0.37-0.61)  1.00  1.41 (1.10-1.79)  1.58 (1.25-2.00)  1.00  1.16 (0.93-1.46)  1.47 (1.18-1.84)  1.00  0.92 (0.73-1.18)  0.96 (0.74-1.25) | Age, sex, race, smoking pack-years, height, alcohol consumption (g/d), triglycerides, and total cholesterol |
| Stoekenbroek RM et al, 2016, United Kingdom | EPIC-Norfolk | 1993-1997 - 2008, 12.1 years follow-up | 21798 men and women, age 45-79 years: 143 abdominal aortic aneurysm cases | AAA | LDL cholesterol | <3.24 mmol/L  3.25-3.88  3.89-4.59  ≥4.60 | 1.00  1.05 (0.64-1.74)  1.12 (0.68-1.84)  1.24 (0.76-2.02) | Age, sex, smoking, BMI, diabetes, HDL cholesterol, systolic blood pressure |
| Kubota Y et al, 2018, USA | Atherosclerosis Risk in Communities study (ARIC) | 1987-2011, 22.6 years follow-up | 13683 men and women, age 45-64 years: 505 abdominal aortic aneurysm cases | AAA | Lipoprotein a | 11μg/ml  29  62  125  246 | 1.00  1.04 (0.79-1.38)  1.15 (0.87-1.52)  1.04 (0.78-1.40)  1.57 (1.19-2.08) | Age, sex, race, height, weight, smoking status, pack-years smoking, hypertension, diabetes mellitus, LDL and HDL cholesterol, triglycerides, and estrogen and/or progesterone use |
| Fattahi N et al, 2020, Sweden | Westmannia Cardiovascular Risk Factors Study (WICTORY) & Västmanland County’s AAA | 1990-1999 - 2007, 15 years follow-up | 5817 men, age 50 years: 153 abdominal aortic aneurysm cases | AAA | Total cholesterol | Per mmol/L | 1.275 (1.119-1.451) | Age, smoking status, blood pressure treatment, prior myocardial infarction, waist circumference, systolic blood pressure |
| Chou EL et al, 2021, USA | Women's Health Initiative | 1993-1998 - 2017, 10.4 years follow-up | 6615 women, age 50-79 years: 415 abdominal aortic aneurysm cases | AAA | Lipoprotein a | 3.5 mg/dl  10.0  18.0  33.0  68.0 | 1.00  1.00 (0.66-1.50)  1.13 (0.75-1.17)  1.30 (0.86-1.96)  1.31 (0.87-1.97) | Race/ethnicity, body mass index, smoking status, pack-years of smoking, a history of hypertension, treated diabetes, and/or CVD; levels of LDL cholesterol(log-transformed), HDL cholesterol (log-transformed),and triglycerides (log-transformed); and MHT use (ie, never used, previous estrogen alone, previous estrogen plus progesterone, current estrogen alone, current estro-gen plus progesterone), statin use, metformin use, and physical activity. |
| Xiao J et al, 2021, Sweden | Malmö Diet and Cancer Study | 1991-1996 - 2016, 19.8 years follow-up | 26688 men and women, age 45-73 years: 447 abdominal aortic aneurysm cases | AAA | Apolipoprotein A1  Apolipoprotein B | Per 2.7-fold change  Per 2.7-fold change | 0.15 (0.09-0.26)  3.54 (2.30-5.46) | Age, sex, sex, marriage status, education, smoking, diabetes, waist, systolic blood pressure, antihypertensive medication, ApoB/ApoA1 ratio, antilipid medication, and white blood cell count. ApoA1 |
| Acosta S et al, 2022, Sweden | Malmö Diet and Cancer Study | 1991-1994, 23.1 years follow-up | 5381 men and women, age 45-73 years: 44 isolated abdominal aortic aneurysm cases | AAA | Total cholesterol | Per 1 mmol/L | 1.12 (0.81-1.55) | Age years, male sex, body mass index, history of hypertension, history of diabetes, current smoking |
| Cho IY et al, 2023, Korea | Korean National Health Insurance Service database | 2009 - 2019, 9.4 years follow-up | 4162640 men and women, age ≥50 years: 18160 abdominal aortic aneurysm cases | AAA | HDL cholesterol  Triglycerides | <50/<40 mg/dL (women/men)  ≥150 mg/dL | 1.46 (1.41-1.50)  1.26 (1.23-1.30) | Age, sex, smoking, alcohol drinking, regular exercise, income, BMI |
| Koba A et al, 2023, Japan | Ibaraki Prefectural Health Study | 1993 - 2019, 26 years follow-up | 95723 men and women, age 40-79 years: 190 aortic aneurysm deaths | AA | Serum HDL cholesterol  Serum non-HDL cholesterol  Serum triglycerides | <40 mg/dL  40-<50  50-<60  ≥60  Per 14.5 mg/dL  <130 mg/dL  130-<150  150-<170  ≥170  Per 35.8 mg/dL  <100 mg/dL  100-<150  150-<200  ≥200  Per 0.52 mg/dL | 2.34 (1.40-3.92)  2.04 (1.32-3.15)  1.34 (0.86-2.09)  1.00  0.71 (0.58-0.86)  1.00  1.03 (0.63-1.66)  1.59 (1.00-2.51)  2.18 (1.40-3.41)  1.44 (1.23-1.68)  1.00  1.01 (0.67-1.50)  0.90 (0.56-1.43)  0.68 (0.41-1.13)  0.84 (0.69-1.01) | Age, sex, systolic blood pressure, diastolic blood pressure, antihypertensive medication, HDL cholesterol, non-HDL cholesterol, triglycerides, lipid-lowering medication, serum glucose, smoking status and cigarettes/day, alcohol |
| Thomas PE et al, 2023, Denmark | Copenhagen General Population Study | 2003-2015 - 2018, 7.4 years follow-up | 70317 men and women, age 20-100 years: 652 abdominal aortic aneurysm cases | AAA | Lipoprotein(a) | <9 mg/dl  10-29  30-94  95-142  ≥143 | 1.00  1.14 (0.94-1.38)  1.36 (1.12-1.65)  1.67 (1.17-2.38)  2.22 (1.21-4.07) | Age, sex, non-HDL cholesterol corrected for Lp(a) cholesterol, systolic blood pressure, smoking status, years of education, diabetes mellitus, BMI, women: menopausal status, and hormone replacement therapy |
| Current study | UK Biobank | 2006-2010 - 2021, 12.3 years follow-up | 429891 men and women, age 38-73 years: 2434 aortic aneurysm cases | AA (also AAA, TAA, UAA) | Total cholesterol  LDL cholesterol  Apolipoprotein B  Non-HDL cholesterol  Triglycerides  Lipoprotein A  HDL cholesterol  Apolipoprotein A1 | 4.393 mmol/l  5.158  5.714  6.295  7.196  2.558 mmol/l  3.135  3.567  4.020  4.713  0.756 g/L  0.910  1.029  1.154  1.350  3.002 mmol/L  3.702  4.237  4.804  5.678  0.787 mmol/l  1.118  1.471  1.956  2.981  4.372 mg/dl  6.830  11.344  24.133  61.573  1.020 mmol/l  1.232  1.413  1.624  1.972  1.226 g/L  1.391  1.520  1.663  1.910 | 1.00  1.12 (0.99-1.27)  1.10 (0.97-1.26)  1.14 (0.99-1.30)  1.22 (1.07-1.41)  1.00  1.14 (1.00-1.30)  1.25 (1.09-1.43)  1.30 (1.13-1.49)  1.39 (1.21-1.60)  1.00  1.14 (1.00-1.30)  1.28 (1.12-1.46)  1.23 (1.07-1.42)  1.52 (1.33-1.74)  1.00  1.18 (1.03-1.35)  1.28 (1.11-1.47)  1.31 (1.13-1.51)  1.50 (1.29-1.73)  1.00  1.18 (1.01-1.37)  1.09 (0.94-1.27)  1.11 (0.96-1.29)  1.23 (1.06-1.42)  1.00  1.04 (0.90-1.20)  1.02 (0.88-1.19)  1.19 (1.03-1.37)  1.34 (1.17-1.54)  1.00  0.84 (0.75-0.94)  0.68 (0.60-0.77)  0.65 (0.56-0.74)  0.57 (0.48-0.67)  1.00  0.86 (0.77-0.96)  0.62 (0.55-0.71)  0.65 (0.57-0.75)  0.54 (0.46-0.63) | Age, sex, ethnicity, Townsend deprivation index, education, smoking status and cigarettes/day, BMI, physical activity, height, hypertension, lipid-lowering medication use |

Abbreviations: AA, aortic aneurysm; AAA, abdominal aortic aneurysm; CI, confidence interval; HDL, high-density lipoprotein; HR, Hazard ratio; LDL, low-density lipoprotein; OR, odds ratio; RR, relative risk; TAA, thoracic aortic aneurysm; UAA, unspecified aortic aneurysm

*The upper confidence interval for the estimate for total cholesterol for the study by Reed (1992) was re-calculated because the confidence intervals were not symmetrical and by comparison with results for other variables it seemed most likely that it was the upper confidence interval was incorrect.

**Supplementary Table 16. Newcastle-Ottawa scale assessment of study quality**

| Author, publication year | Selection | | | Comparability | Outcome assessment | | | Total |
| --- | --- | --- | --- | --- | --- | --- | --- | --- |
|  | Selection of non-exposed cohort | Exposure ascertainment | Demonstration of outcome not present at start | 0.25 points for each adjustment | Outcome assessment | Long enough follow-up (≥5 years) | Adequacy of follow-up (≤10% lost) |  |
| Strachan DP et al, 1991 | 1 | 1 | 0 | 1.25 | 1 | 1 | 0 | 5.25 |
| Reed D et al, 1992 | 1 | 1 | 1 | 0.75 | 1 | 1 | 1 | 6.75 |
| Goldberg RJ et al, 1995 | 1 | 1 | 1 | 2 | 1 | 1 | 1 | 8.00 |
| Lee AJ et al, 1997 | 1 | 1 | 0 | 1.25 | 1 | 1 | 0 | 5.25 |
| Watt HC et al, 1998 | 1 | 1 | 0 | 1 | 1 | 1 | 0 | 5.00 |
| Törnwall MF et al, 2001 | 1 | 1 | 1 | 2 | 1 | 1 | 1 | 8.00 |
| Rodin MB et al, 2003 | 1 | 1 | 1 | 2 | 1 | 1 | 1 | 8.00 |
| Lindblad B et al, 2005 | 1 | 1 | 1 | 0.25 | 1 | 1 | 1 | 6.25 |
| Wanhainen A et al, 2005 | 1 | 1 | 0 | 0.50 | 1 | 1 | 1 | 5.50 |
| Iribarren C et al, 2007 | 1 | 1 | 0 | 2 | 1 | 1 | 0 | 6.00 |
| Forsdahl SH et al, 2009 | 1 | 1 | 1 | 0.50 | 1 | 1 | 0 | 5.50 |
| Sode BF et al, 2013 | 1 | 1 | 1 | 2 | 1 | 1 | 1 | 8.00 |
| Sode BF et al, 2013 | 1 | 1 | 1 | 2 | 1 | 1 | 1 | 8.00 |
| Landenhed M et al, 2015 | 1 | 1 | 1 | 0.75 | 1 | 1 | 0 | 5.75 |
| Tang W et al, 2016 | 1 | 1 | 1 | 2 | 1 | 1 | 1 | 8.00 |
| Stoekenbroek RM et al, 2016 | 1 | 1 | 0 | 1.75 | 1 | 1 | 0 | 5.75 |
| Kubota Y et al, 2018 | 1 | 1 | 1 | 2.00 | 1 | 1 | 1 | 8.00 |
| Fattahi N et al, 2020 | 1 | 1 | 1 | 1.75 | 1 | 1 | 1 | 7.75 |
| Chou EL et al, 2021 | 1 | 1 | 1 | 2.00 | 1 | 1 | 1 | 8.00 |
| Xiao J et al, 2021 | 1 | 1 | 1 | 2.00 | 1 | 1 | 1 | 8.00 |
| Acosta S et al, 2022 | 1 | 1 | 1 | 1.50 | 1 | 1 | 1 | 7.50 |
| Cho IY et al, 2023 | 1 | 1 | 1 | 1.75 | 1 | 1 | 0 | 6.75 |
| Koba A et al, 2023 | 1 | 1 | 0 | 2.00 | 1 | 1 | 1 | 7.00 |
| Thomas PE et al, 2023 | 1 | 1 | 0 | 2.00 | 1 | 1 | 0 | 6.00 |
| Current study | 1 | 1 | 1 | 2.00 | 1 | 1 | 1 | 8.00 |

**Supplementary text. Search strategy in PubMed and Embase**

**PubMed:**

1) (cholesterol OR lipid OR triglyceride OR LDL OR HDL OR apolipoprotein OR lipoprotein OR dyslipidemia)

2) (aortic aneurysm OR thoracic aneurysm OR abdominal aneurysm)

3) #1 AND #2

**Embase:**

1) (cholesterol or lipid* or triglyceride* or LDL or HDL or apolipoprotein or lipoprotein* or dyslipedemia).ab,ti.

2) cholesterol/ or lipid*/ or triglyceride*/ or LDL/ or HDL/ or apolipoprotein*/ or lipoprotein*/ or dyslipidemia/

3) (aortic aneurysm OR thoracic aneurysm OR abdominal aneurysm).ab,ti.

4) aortic aneurysm/ OR thoracic aneurysm/ OR abdominal aneurysm/

5) #1 or #2

6) #3 or #4

7) #5 and #6

**Supplementary Figure 1. Influence analysis of total cholesterol and aortic aneurysm**

------------------------------------------------------------------------------

Study omitted | e^coef. [95% Conf. Interval]

-------------------+----------------------------------------------------------

UKB | 1.1803371 1.1069772 1.2585585

Acosta, 2022 | 1.1593449 1.0978092 1.2243299

Fattahi, 2020 | 1.146829 1.0869212 1.2100387

Tang, 2016 | 1.1805667 1.1068453 1.2591983

Forsdahl, 2009 | 1.1728051 1.1043564 1.2454963

Iribarren, 2007 | 1.1470958 1.0870725 1.2104332

Lindblad, 2005 | 1.1508045 1.0913221 1.213529

Wanhainen, 2005 | 1.1459165 1.0892884 1.2054886

Rodin, 2003 | 1.1417674 1.0837523 1.2028883

Tornwall, 2001 | 1.1696953 1.102107 1.2414284

Watt, 1998 | 1.1580726 1.0968201 1.2227459

Lee, 1997 | 1.1579819 1.0963297 1.223101

Goldberg, 1995 | 1.1610681 1.0971534 1.228706

Reed, 1992 | 1.1356299 1.0806396 1.1934186

Strachan, 1991 | 1.1680994 1.1059027 1.2337942

-------------------+----------------------------------------------------------

Combined | 1.1578182 1.0976543 1.2212799

------------------------------------------------------------------------------

**Supplementary Figure 2. Funnel plot for total cholesterol and aortic aneurysm**

**Supplementary Figure 3. Total cholesterol and aortic aneurysm, high vs. low analysis**

**Supplementary Figure 4. LDL cholesterol and aortic aneurysm, high vs. low analysis**

**Supplementary Figure 5. Apolipoprotein B and aortic aneurysm, high vs. low analysis**

**Supplementary Figure 6. Non-HDL cholesterol and aortic aneurysm, high vs. low analysis**

**Supplementary Figure 7. Influence analysis of HDL cholesterol and aortic aneurysm**

------------------------------------------------------------------------------

Study omitted | e^coef. [95% Conf. Interval]

-------------------+----------------------------------------------------------

UKB | 0.60383725 0.4753218 0.76710021

Koba, 2023 | 0.56292462 0.46667472 0.67902571

Tang, 2016 | 0.56140518 0.45901927 0.68662864

Forsdahl, 2009 | 0.5934186 0.48262227 0.72965056

Tornwall, 2001 | 0.63093585 0.54234529 0.73399734

-------------------+----------------------------------------------------------

Combined | 0.59077112 0.49586291 0.70384476

------------------------------------------------------------------------------

**Supplementary Figure 8. HDL cholesterol and aortic aneurysm, high vs. low analysis**

**Supplementary Figure 9. Apolipoprotein A1 cholesterol and aortic aneurysm, dose-response analysis per 1 g/L**

**Supplementary Figure 10. Apolipoprotein A1 cholesterol and aortic aneurysm, high vs. low analysis**

**Supplementary Figure 11. Influence analysis of triglycerides and aortic aneurysm**

------------------------------------------------------------------------------

Study omitted | e^coef. [95% Conf. Interval]

-------------------+----------------------------------------------------------

UKB | 1.024716 0.91171396 1.1517242

Koba, 2023 | 1.0440037 0.97352844 1.1195807

Tang, 2016 | 1.0350274 0.95094877 1.1265398

Lindblad, 2005 | 1.0327264 0.92167121 1.1571631

Wanhainen, 2005 | 1.0188326 0.96654314 1.0739508

Goldberg, 1995 | 1.0283544 0.94840449 1.1150441

Reed, 1992 | 1.0125855 0.93901742 1.0919174

-------------------+----------------------------------------------------------

Combined | 1.023569 0.95029796 1.1024895

------------------------------------------------------------------------------

**Supplementary Figure 12. Funnel plot of triglycerides and aortic aneurysm**

**Supplementary Figure 13. Triglycerides and aortic aneurysm, high vs. low analysis**

**Supplementary Figure 14. Lipoprotein(a) and aortic aneurysm, high vs. low analysis**
